# The development of early pioneer neurons in the annelid *Malacoceros fuliginosus*

**DOI:** 10.1186/s12862-020-01680-x

**Published:** 2020-09-14

**Authors:** Suman Kumar, Sharat Chandra Tumu, Conrad Helm, Harald Hausen

**Affiliations:** 1 Sars International Centre for Marine Molecular Biology, University of Bergen, Bergen, Norway; 2 Present Address: Johann-Friedrich-Blumenbach Institute for Zoology and Anthropology, Georg-August-Universität Göttingen, Göttingen, Germany

**Keywords:** Nervous system, Development, Evolution, Annelida, Pioneer neurons

## Abstract

**Background:**

Nervous system development is an interplay of many processes: the formation of individual neurons, which depends on whole-body and local patterning processes, and the coordinated growth of neurites and synapse formation. While knowledge of neural patterning in several animal groups is increasing, data on pioneer neurons that create the early axonal scaffold are scarce. Here we studied the first steps of nervous system development in the annelid *Malacoceros fuliginosus*.

**Results:**

We performed a dense expression profiling of a broad set of neural genes. We found that *SoxB* expression begins at 4 h postfertilization, and shortly later, the neuronal progenitors can be identified at the anterior and the posterior pole by the transient and dynamic expression of proneural genes. At 9 hpf, the first neuronal cells start differentiating, and we provide a detailed description of axonal outgrowth of the pioneer neurons that create the primary neuronal scaffold. Tracing back the clonal origin of the ventral nerve cord pioneer neuron revealed that it is a descendant of the blastomere 2d (2d^221^), which after 7 cleavages starts expressing *Neurogenin*, *Acheate-Scute* and *NeuroD*.

**Conclusions:**

We propose that an anterior and posterior origin of the nervous system is ancestral in annelids. We suggest that closer examination of the first pioneer neurons will be valuable in better understanding of nervous system development in spirally cleaving animals, to determine the potential role of cell-intrinsic properties in neuronal specification and to resolve the evolution of nervous systems.

## Background

The basic scheme of early neurogenesis, that is, the early steps in the development of individual neurons, depends in eumetazoans on a fairly conserved set of transcription factors, many of them belonging to the *Sox* and proneural *bHLH* gene families [1, 2]. Though regulation and interdependency display variability between groups, these genes were in many organisms found to play important roles in neurogenesis: from providing neurogenic potential in the ectoderm of early embryos and controlling the step-wise transition of self-renewing multi-potential neuronal progenitors to more committed precursors which later differentiate into specific neurons or glia elements [3–6]. Where, when, and which neuronal types are formed depends on patterning processes, which are intimately linked to the above-described steps of neurogenesis and the subsequent process of differentiation, like broad anterior-posterior and dorso-ventral patterning of the ectoderm and more local patterning processes during subdomain development [7–11].

Yet nervous system architecture differs considerably in the different evolutionary lineages of bilaterian animals [12]. Many of them possess a centralized nervous system comprising of a central brain and longitudinal trunk nerve cords. But brain structure and the arrangement, number, branching, and organization of the nerve cords vary considerably between taxa, and some groups display only a low level of nervous system centralization and instead exhibit diffuse nerve nets. It is still intensely debated how conserved are the main processes of nervous system patterning in bilaterians and how nervous system centralization evolved, whether it is homologous or arose independently in different lineages [13–18]. In this context, studies in the protostome group of lophotrochozoans received attention during the last years. While data on neurogenesis and nervous system pattering from some representatives suggest high similarity to vertebrates and arthropods [19–27], the nervous system architecture is quite diverse within the group and scenarios of common ancestry as well as independent evolution of nervous system centralization have been claimed [13, 15].

One important aspect of nervous system development, which has hardly been addressed in these animals, is the specification and specific role of the pioneer neurons, which prefigure the primary scaffold of the central nervous system (CNS). Comparative immunohistochemical studies on early nervous system development in lophotrochozoans provide conflicting data on whether the nervous system forms from the anterior or the posterior pole or both [28, 29]. This may partly result from the fact that only a small subset of neurons is stained by standard markers for certain neurotransmitters, but also from diversity, which is also obvious in the adult nervous system organization [12, 30]. Within annelids, nervous system development is best characterized in *Platynereis dumerilii,* representing the large subgroup of errant polychaetes and *Capitella teleta*, representing the sedentary subgroup. The data differ on the origin of neural structures (anterior and posterior in *P. dumerilli* and only anterior in *C. teleta*), the patterning along the anterior-posterior, and the mediolateral axes [31, 32]. We chose the sedentary polychaete *Malacoceros fuliginosus* as a study subject due to its experimental suitability and to find answers on the ancestral mode of early nervous development in annelids and beyond.

We describe for the first time in an annelid the very first steps of neurogenesis. We find that the pioneer neurons are already in place very early when neurogenesis and patterning of the vast majority of neurons are yet to start. Expression of proneural genes starts already at 5 h postfertilization and after only a few rounds of embryonic cleavages. The early expression is restricted to very few cells at the anterior and posterior pole. The expression in the early stages is very dynamic and punctuated before expanding in specific areas of neuronal proliferation. A single posterior neuron prefigures the main course of the ventral nerve cord (VNC),while in the anterior region, the central ganglia is initiated by a single apical neuron and a pair of sensory cells giving rise to the prototroch nerve ring. Due to its position, we could track the development of the posterior neuron in detail from early specification to the onset of differentiation and outgrowth of the neurites. From our data, an anterior and posterior origin of the nervous system is likely ancestral for the majority of annelids and possibly beyond. The early specification of pioneer neurons makes it interesting subjects for studies on neural specification, the role of cell-intrinsic and extrinsic factors on the development, and the evolutionary conservation of nervous system development.

## Results

### The first pioneer neurons with long neurites appear at the anterior and the posterior pole of the larvae

**Fig. 1:**
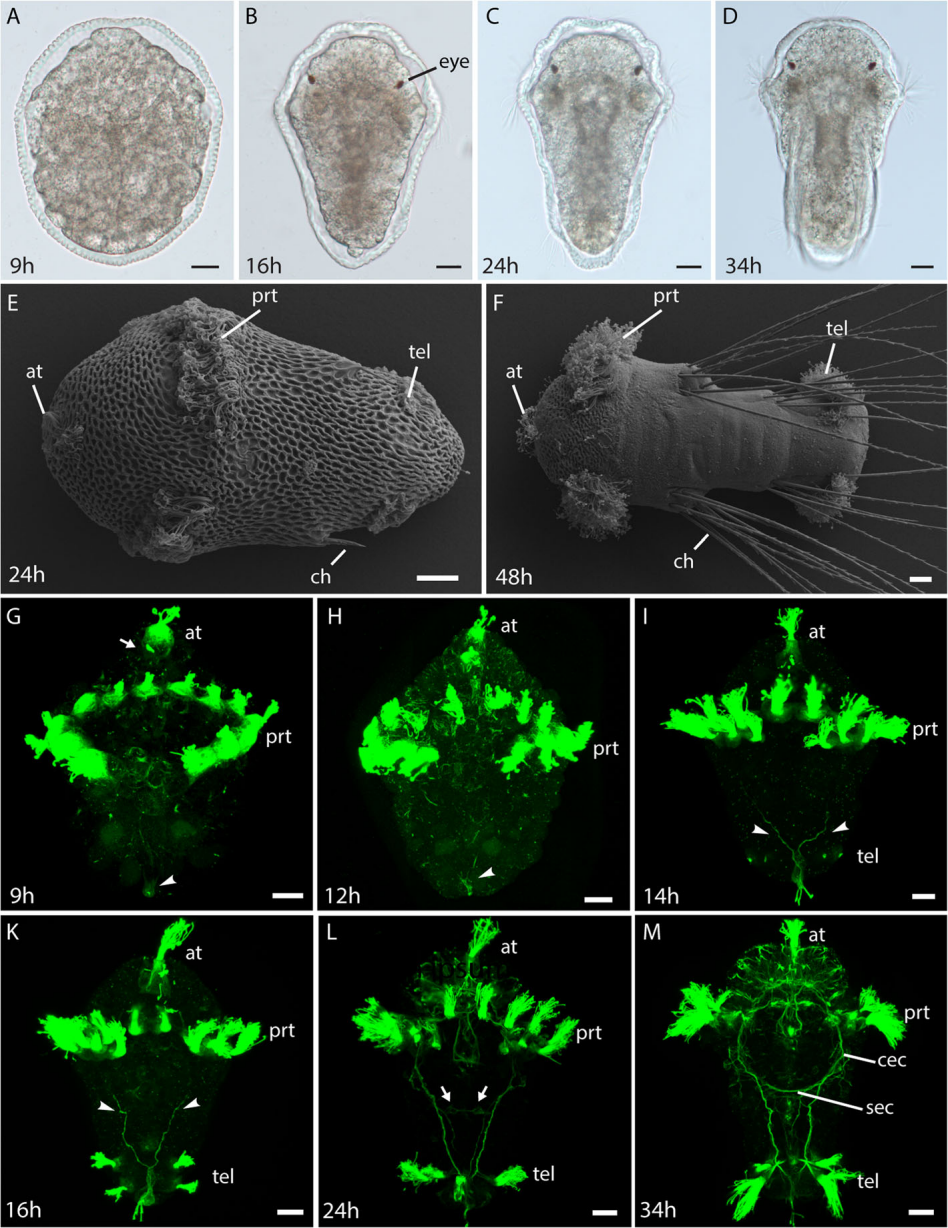
Development of early larval stages of *Malacoceros fuliginosus*. **a**-**d** Light microscopic images of larval development. **e**, **f** SEM images showing the dorsal view of *Malacoceros fuliginosus* larva. **g**-**m** Nervous system development in *Malacoceros fuliginosus* as revealed by acetylated α-tubulin (ac-tubulin) immunolabeling (green). The primary ciliated structures such as apical tuft (at), prototroch (prt) and telotroch (tel) are also revealed. The arrowheads point to the developing posterior pioneer neuron (PPN) and bifurcating axons. Arrows point to the first ventral nerve cord commissure. Scale bars: 20 μm. ch: chaetae

The early development of the *M. fuliginosus* larva is highly synchronous and therefore allows for precise staging across batches. Throughout the early development up to stages of 24 hpf, the larva is surrounded by a thick ornamented chorion of the egg (Fig. 1a-d, e). The chorion later becomes an integral part of the cuticle, which at 48 hpf already has a smooth surface (Fig. 1f). Cilia of the prototroch and the apical tuft penetrate the chorion from 7 hpf onwards. Pigmented eyes appear at around 14 hpf (Fig. 1b-d), and chaetae start penetrating the chorion around 24 hpf (Fig. 1e) and form prominent bundles at 48 hpf (Fig. 1f). Immunohistochemistry shows that the prototroch and the telotroch are formed by bundles of cilia (Fig. 1g-m), which in later stages is less obvious by external examination (Fig. 1e, f). The prototroch band is not continuous on the dorsal body surface from the time of its emergence (Fig. 1e-i). The nervous system development is relatively fast, and due to a low yolk content of the larvae, a detailed investigation is possible by immunohistochemical stainings. For this purpose, we performed antibody stainings against acetylated alpha-tubulin (a-tub) from 7 hpf onwards, where only the cilia of the prototroch and an apical tuft are visible.

**Fig. 2:**
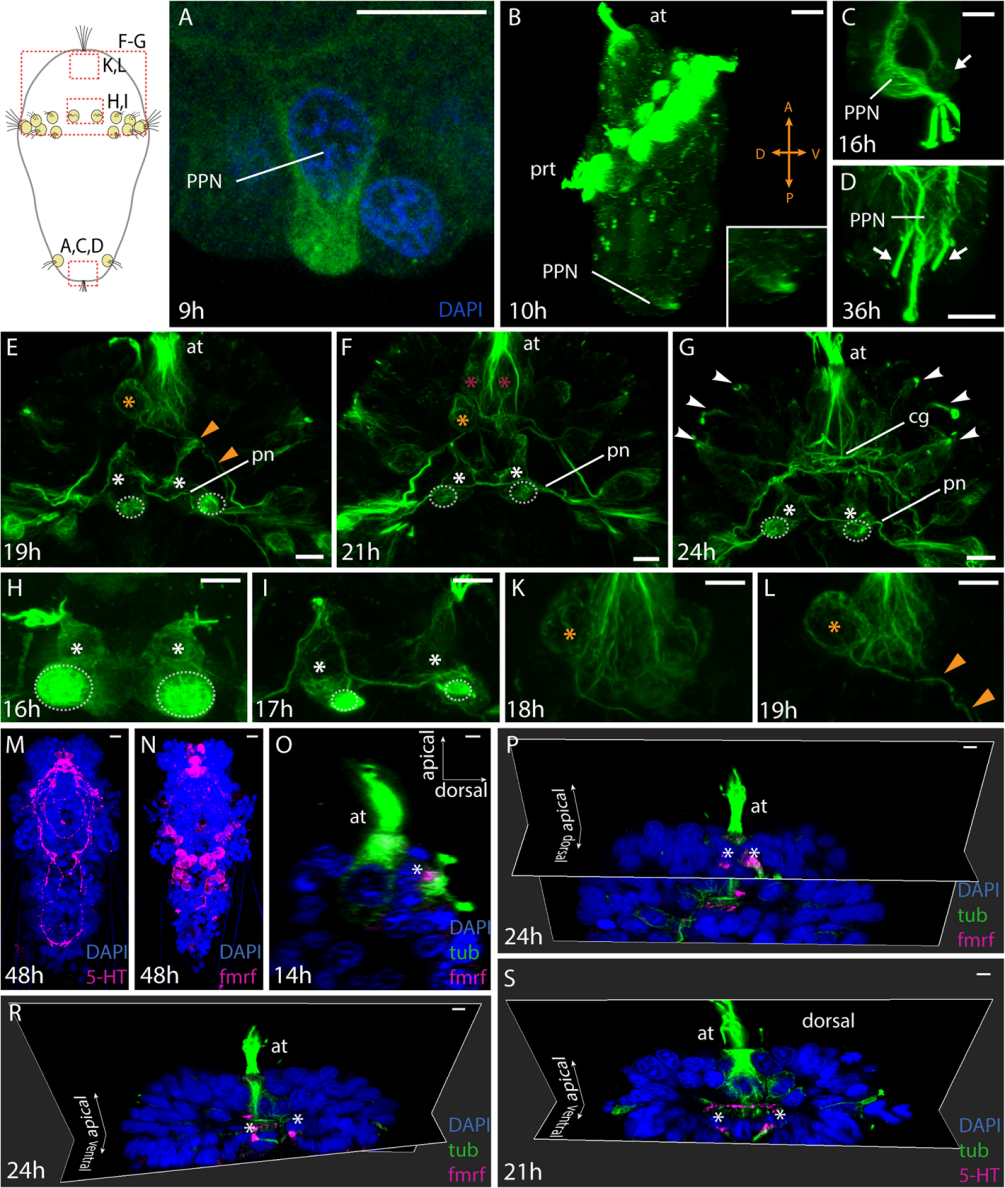
Pioneer neurons forming the main neuronal scaffold. The schematic diagram indicates the larval regions shown in the figures. **a**-**d** Posterior pioneer neuron (PPN). **a** Dense tubulin marks the soma of the posterior pioneer neuron (PPN) already at 9 hpf. **b** Lateral view at 10 hpf showing the orientation of the PPN (inset). **c** A single dorsal follower neuron of the PPN is visible from 16hpf. **d** A lateral pair of follower neurons of the PPN at 36hpf. **e**-**g** Development of the anterior neuronal scaffold. An apical ganglion cell (AP1) (orange asterisk) on the left body side sends a descending axon towards the right body side. A pair of sensory cells (white asterisk) give rise to the prototroch nerve ring. The dotted circles show the adjacent prototroch cells. Neurites from later appearing neuronal pairs (red asterisk) and peripheral sensory neurons (arrowheads) converge to form the central neuropil. **h**, **i** Detailed few the pioneers of the prototroch nerve ring. **k**, **l** Detailed view of the apical ganglion cell AP1. **m**-**s** Immunohistochmical staining of serotonin (5-HT) and FMFRamide during early nervous system development. **m**, **n** 5-HT and FMRFamide are detected in many neurons and neurites at 48 h. **o** First FMRFamide is detected at 14 hpf in a ciliated sensory cell dorsal and left of the apical tuft. **p** Dorsal apical pair of FMRFamidergic cells. **r**, **s** One FMRFamidergic and one serotogener apical pair of ciliated sensory cells ventral of the apical tuft. Asterisks (**o**-**s**): nucleus of cells with FMRFamide or 5-HT. Scale bars: 10 μm, (except F: 20 μm, N-Q: 5 μm). at: apical tuft, cec: circumesophageal connective, cg: central ganglia, prt: prototroch, sec: supraesophageal connective, tel.: telotroch

**Fig. 3:**
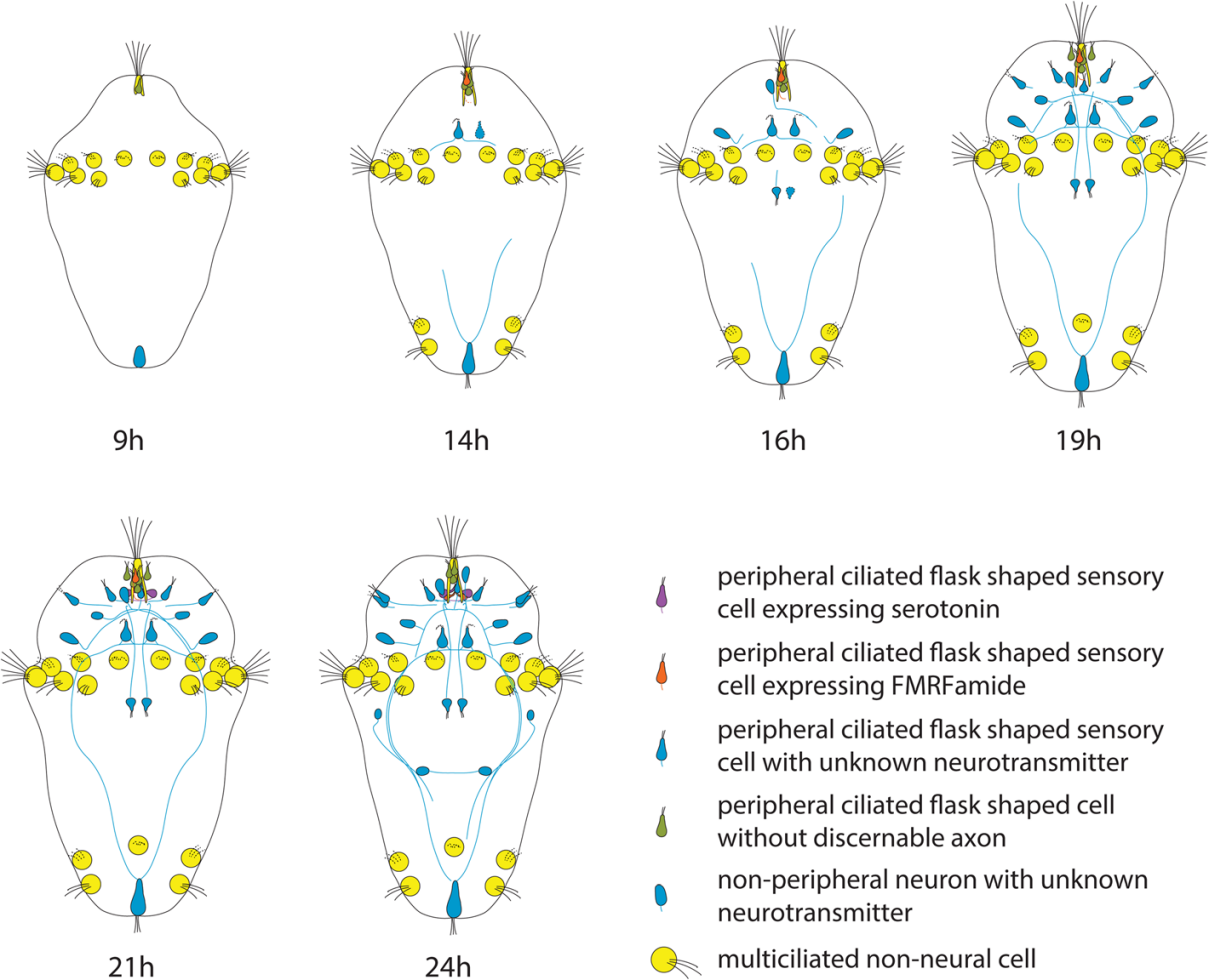
Schematic reconstruction of early nervous system development based on ac-tub, 5-HT and FMRFamide, and nuclear stainings

Studying stages in short time intervals allowed us to identify the first appearing pioneer neurons that send out the first neurites and initiate the formation of the early neuronal scaffold. The first neuron to send out axonal processes is a single posterior pioneer neuron (PPN), which starts to differentiate around 8 hpf with the accumulation of dense microtubules (data not shown) and acquires a distinct morphology by 9 hpf (Figs. 1g, 2a, 3, Additional file 1). This bifurcating cell projects two axons proceeding towards the prototroch from 12 hpf onwards (Fig. 1h), which extends already one third to the prototroch at 14 hpf (Figs. 1i, 3, Additional files 2, 3, 4). During this phase, the PPN also acquires a curved morphology (Fig. 2b) with few sensory cilia extending outwards on the ventral side. The axons of the PPN reach the prototroch area around 19 hpf (Additional files 8, 9, Fig. 3). At around 16 hpf, a new neuron (without sensory cilia) and presumably a follower develops adjacent to the PPN (Fig. 2c). It extends axons along the processes formed by the PPN (Additional files 5, 6, 7). More follower neurons appear at 34 hpf, when a pair of weakly stained ciliated sensory cells are visible on either side of the PPN and become more prominent at 36 hpf (Fig. 2d).

In the anterior end, the first neurons can be observed from 9 hpf underneath the apical tuft (Figs. 1g, 3, Additional file 1), but we could not identify extending axons before 14 hpf. At this time, several cells lie underneath the apical tuft, but only one sends a short axon towards the prospective brain neuropil (Fig. 3, Additional files 2, 3, 4). At the same time, the prototroch nerve starts to develop from a pair of cells situated adjacent to the ventromedial prototroch cells. We name these cells as the prototroch nerve forming neurons (PNNs). These cells have a triangular morphology and sensory cilia projecting externally. Interestingly, for a few hours, only one of these cells sends out processes extending on either side along the prototrochal cells (Figs. 2h, i, 3, Additional files 5, 6, 7). From 19 hpf onwards, the other cell, which only has thin neurites projects onto the passing connectives from the adjacent cell (Figs. 2e, f, 3, Additional files 8, 9, 10, 11, 12). On the other hand, the surrounding prototroch cells seem to send extensions towards the prototroch nerve (Additional files 5, 6, 7, 8, 9, 10, 11, 12). As the prototroch is discontinuous on the dorsal side, the prototroch nerve extends only until the dorsal-most prototroch cell without forming a complete ring.

At 16 hpf, a single ganglion cell appears on one side of the apical organ along with a descending axon (Fig. 3, Additional files 5, 6, 7), which becomes more prominent at 18 hpf (Fig. 2k). We name this pioneer as the apical neuron 1 (AN1). Around 19 hpf, this growing axon from AN1 traverses contralaterally up to the prototroch region (on the other side). It extends posteriorly towards the anteriorly traveling neurite of the PPN (Figs. 2l, 3, Additional files 8, 9) and thereby closing the gap between anterior and posterior parts of the developing VNC. Meanwhile, at the same time, the growing axons from PNNs travel posteriorly and join the developing VNC (Fig. 2e).

At 21 hpf, more cells appear symmetrically around the apical organ and traverse contralaterally along the neurites established by the AN1, which persists (Figs. 2f, 3, Additional files 10, 11, 12). The crisscross of neurites originating alongside the apical organ creates a plexus, which becomes the larval brain neuropil (Figs. 2f, 3). By 24 hpf, the AN1 becomes less prominent and is not easily identifiable as more and more differentiated cells start to innervate the brain neuropil (Figs. 2g, 3, Additional files 13, 14, 15, 16). The PNNs, however, are still identifiable at 24 hpf (Fig. 2g), which by 34 hpf becomes less conspicuous (data not shown).

Around 24 hpf, a weakly stained commissure is arising in the anterior trunk region that connects the two VNC neurite bundles (Figs. 1l, 3, Additional files 13, 14, 15, 16). The nervous system represented by the 34 hpf stage is of a typical annelid trochophore composed of an apical tuft, prototroch, telotroch, and neuronal elements such as central cephalic neuropil, a prototroch nerve ring (semi-circular) and VNC (Fig. 1m). This basic neuronal architecture continues into the later larval stages and likely becomes part of the adult nervous system. In the anterior end, numerous sensory cells develop throughout the head region, with projections reaching the brain neuropil (Fig. 1m).

### Development of serotonergic and FMRFamidergic neurons

For comparative purposes, we studied immunoreactivity against serotonin (5-HT) and FMRFamide, which are commonly used markers in studies on invertebrate neural development. 5-HT and FMRF are important neurotransmitters in many animals [33, 34]. While 5-HT and FMRFamide are strongly expressed in the apical neuropil and the trunk in 48 hpf stages (Fig. 2m, n), only a few serotonergic and FMRFamidergic cells are present in earlier stages. FMRFamide is detectable first at 14 hpf as a single, ciliated flask-shaped weakly labeled cell, which lies slightly left and dorsal from the apical tuft cell (Figs. 2o, 3, Additional files 2, 3). This cell develops an axon that is later projecting into the area of the apical neuropile and is accompanied by a similar second cell on the right body side at 24 hpf (Figs. 2p, 3, Additional files 14, 16). Already at 21 hpf, a second pair of FMRFamidergic ciliated flask-shaped cells also sending axons in the apical neuropil become visible ventrally from the apical tuft cell (Fig. 3, Additional file 11) and are visible as well at 24 hpf (Figs. 2r, 3, Additional files 14, 16). 5-HT can be detected from 21 hpf onwards in a pair of cells ventral to the apical tuft cell, which likewise are ciliated and flask-shaped and send their axons into the forming apical neuropil (Figs. 2s, 3, Additional file 10). Up to the 24 hpf stage, neither -FMRFamide nor 5-HT is detectable in the mid or posterior body region. In all investigated stages, only a very small fraction of the identified neurons and none of the described neurons pioneering the ventral nerve cord, the prototroch, or the connection between the apical plexus and the VNC contains FMRFamide or 5-HT.

### The first neurons show synaptic activity likely from 12 hpf onwards

To get an idea when the developing neurons are entering differentiation and are getting functional on the molecular level, we screened the transcriptome resources of *M. fuliginosus* and public sequence databases for orthologs to synaptotagmin-1 (Syt1), which is a conserved Ca^2+^ sensor for fast synaptic vesicle exocytosis in many neurons of metazoans, and ras-related protein 3 (Rab3), which regulates synaptic vesicle fusion. Two sequences were found, which after reciprocal blast against Genbank gave only Syt1 and Rab3 sequences as the first 100 hits, and we named them Mfu-Syt1 and Mfu-Rab3.

In order to find an ortholog for the RNA binding protein Elav1, which is a common marker for postmitotic neuronal precursor cells in many metazoans, we ran a maximum-likelihood tree of metazoan Elav and CUGBP Elav-like genes. As in many other lophotrochozoans, in *M. fuliginosus*, we found two CUGBP Elav-like sequences, and two Elav sequences, both of which have RRM RNA binding motifs. One sequence (Mfu-Elav2) groups with other lophotrochozoan sequences (Additional file 17) confirming the existence of a lophotrochozoan specific *Elav2* gene [21, 35]. The other one (Mfu-Elav1) clusters with high support with metazoan *Elav1*. Since the expression of *Elav2* is not much studied and is not specific to neurons in *Capitella teleta* [21] and *Sepia officinalis* [35], we investigated only the expression of *Mfu-Elav1*.

Similarly, we ran an analysis of metazoan *POU* genes to identify the *M. fuliginosus* ortholog to POU4, which is an important regulator of terminal differentiation in many neurons of Metazoa. We found several POU gene sequence in the transcriptomic resources of *M. fuliginosus*, all containing a POU-specific domain. Only one, *Mfu-POU4*, belongs to the well supported *POU4* clade (Additional file 18). Likewise, most other lophotrochozoans have only one *POU4* gene. If there are more, then they are the result of species-specific gene duplications.

**Fig. 4:**
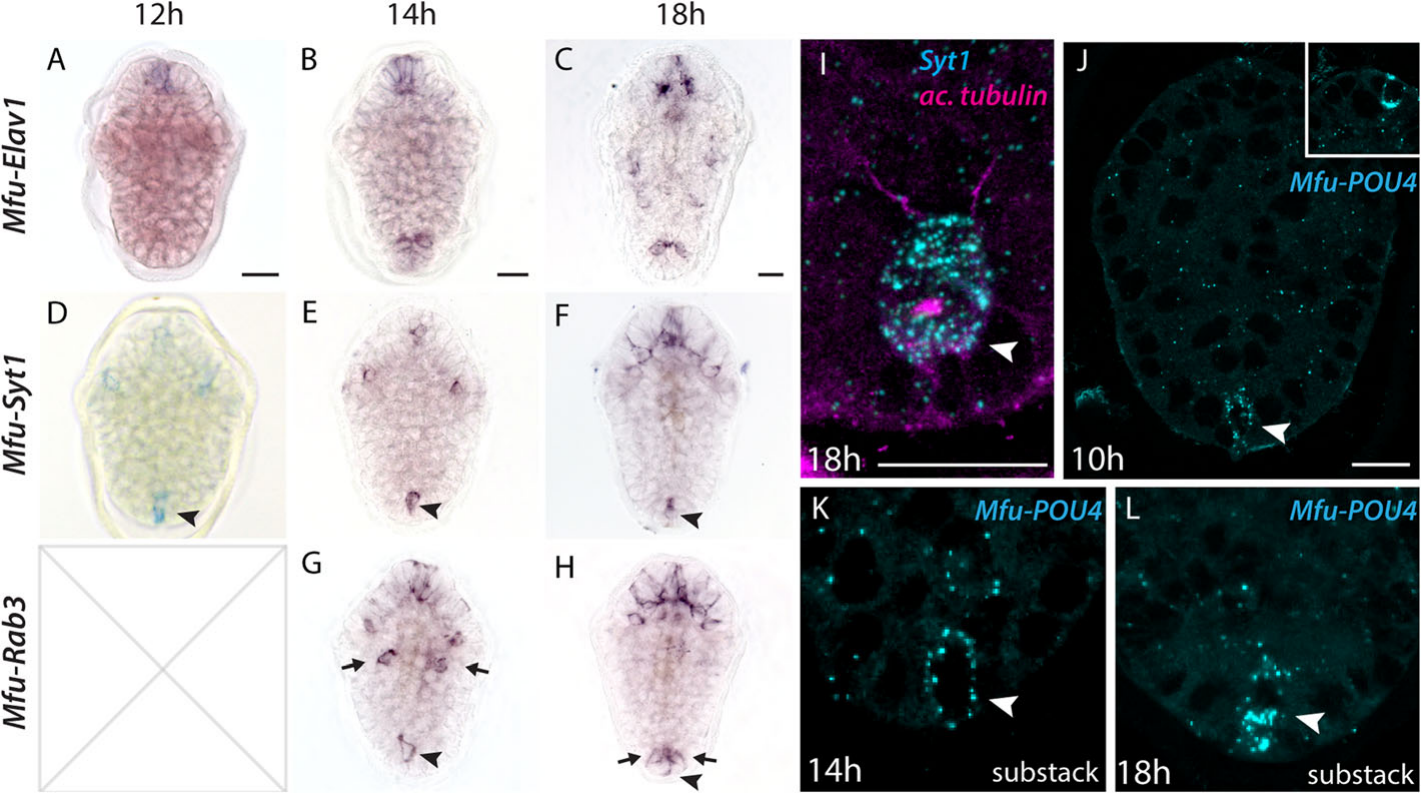
Expression of differentiation genes during larval development. **a**-**h** WISH of neuronal genes *Mfu-Elav1*, *Mfu-Syt1*, *Mfu-Rab3*. **i** FISH of *Mfu-Syt1*combined with IHC of acetylated-tubulin in the posterior region. **j**-**l** FISH of *Mfu-POU4*. Inset in (**j**) shows the expression of *Mfu-POU4* in the anterior region. Arrowheads point to the posterior pioneer neuron, while arrows point to the neurons adjacent to it. Scale bars: 20 μm

*Mfu-Elav1* and *Mfu-Syt1* were first detected at 12hpf (Fig. 4a, d). At this stage, *Mfu-Elav1* is only restricted to the anterior region close to the apical tuft, whereas *Mfu-Syt1* is expressed near the apical region, in a pair of bilateral cells and in the posterior region (Fig. 4a, d). While the expression of *Mfu-Syt1* in the bilateral cells corresponds to the developing eye photoreceptors (data not shown), the posterior region corresponds to the PPN, as shown by co-staining with ac-tubulin (Fig. 4i). Shortly later, at 14 hpf, *Mfu-Elav1* also starts expressing in the posterior region but not in the PPN. At the same stage, *Mfu-Rab3* starts expressing in few cells spanning the anterior, middle, and posterior regions in a similar pattern to *Mfu-Syt1*, nonetheless, in more cells (Fig. 4g). From 18hpf onwards, several cells throughout the body express *Mfu-Elav1*, while *Mfu-Syt1* and *Mfu-Rab3* are mainly restricted to the anterior and posterior regions. The dense staining of *Mfu-Syt1* and *Mfu-Rab3* in the anterior region reflects the many neurons contributing to the central neuropil (Fig. 4f, h). In the posterior region, *Mfu-Syt1* is only expressed in the PPN, whereas *Mfu-Rab3* is expressed in the PPN and cells adjacent to it (Fig. 4h). By 48 hpf, more neurons in the anterior, oral, and along the developing VNC express *Mfu-Syt1* and *Mfu-Rab3* (Additional file 24).

The terminal selector *POU4* (*Mfu-POU4*) starts to express from 10hpf in two cells, one in the anterior region and one in the posterior region (Fig. 4j). The expression in the posterior region very likely corresponds to the PPN as determined by the position and morphology of the cell. At 14hpf, an additional pair of cells lying adjacent to the posterior neuron start to express *Mfu-POU4* (data not shown). In subsequent stages, the expression of *Mfu-POU4* expands mainly in the anterior peripheral cells (from 12hpf) and along the trunk (from 18hpf) (data not shown). The expression of *Mfu-POU4* in the PPN is maintained until the 24 hpf, after which it gets weaker.

### Evolution of *M. fuliginosus Sox, Prospero* and *bHLH* genes

Sox, Prospero, and bHLH genes are important regulators of neurogenesis in many metazoans. Several genes of these groups duplicated and diversified during evolution, and many organisms have several copies of specific genes. To clarify the orthology relationships of the genes we found in the transcriptome resources of *M. fuliginosus,* we performed broad phylogenetic analyses with a focus on a good taxon sampling in Lophotrochozoa. *Prospero1* (*Prox1*) genes are fairly distinct from other homeobox genes. Thus, we chose not to include any outgroup for tree inference. In our unrooted tree, the single *Mfu-Prox1* groups with high support with other lophotrochozoan *Prox1* genes (Additional file 19), and, thus, they are regarded as orthologs. We did not find more than one *Prox1* in any lophotrochozoan.

bHLH genes are a large group of genes sharing the basic helix-loop-helix motif. Many genes of the bHLH subgroup A family: Achaete scute (ASC), Oligo, Beta3, Neurogenin, NeuroD, and Atonal play important roles in neural development. Based on a first analysis, which in addition to the above-mentioned genes includes many other bHLH group A genes, we obtained 6 Achaete scute (ASC), 3 Oligo, one each of Beta3, Atonal, Neurogenin and NeuroD sequences for *M. fuliginosus* (Additional file 20). For gene annotation, we adopted the terminology of [36]. The gene numbers in the specific families are similar as in other lophotrochozoans for which genomic data exist, and all families are well supported in the tree with the exception of *NeuroD* and *Oligo*, which are paraphyletic. Since within this assemblage *Mfu-NeuroD* clusters well-supported with *NeuroD* from other lophotrochozoans, we regard it as being an ortholog to those. Based on our tree, we could not resolve the 1:1 orthologs of the three *M. fuliginosus Oligo* genes. Since not many comparative data exist on *Oligo*, we did not perform further analyses and named the genes *Mfu-OligoA*, *Mfu-OligoB*, and *Mfu-OligoC*. We recorded clear expression patterns for *Mfu-OligoA*, *Mfu-OligoB*, *Mfu-NeuroD*, *Mfu-neurogenin*, and *Mfu-ASCa1*, *Mfu-ASCa2*, *Mfu-ASCa3*, and *Mfu-ASCa4*.

To get insights into the relationship of the *M. fuliginosus ASC* genes to those studied in other species, especially *C. teleta* and *P. dumerilii*, we generated a further tree based on deeper sampling and a more ASC specific and longer alignment. It corroborates the three big metazoan clades *ASCc, ASCb* containing vertebrate *ASC3–5*, and *ASCa* containing arthropod *ASC T1-T8* and vertebrate *ASC1–2* (Additional file 21). *M. fuliginosus* has representatives of all *ASC* groups as do other lophotrochozoans. Four *M. fuliginosus* sequences belong to the clade *ASCa*. The evolution of this group is in lophotrochozoans seemingly more complex than hitherto anticipated, since many species have several gene copies, many of which are poorly studied. *Mfu-ASCa1* and *Mfu-ASCa2* are closely related to *P. dumerilli Achaete-scute 1* and to *C. teleta Achaete scute 2* (Additional file 21), but also to two more transcripts from *P. dumerilii*, which we received from the Genbank TSA database, for which no cellular expression data exist, but according to PdumBase [37] are highly expressed in larvae from early on. To give a final answer on clear orthology relationships amongst these 6 genes is not possible based on our tree. It also remains unclear whether *Mfu-ASCa3* has an ortholog in *P. dumerilii* and *C. teleta*. *Mfu-ASCa4* clusters with another transcript of *P. dumerilii*, which according to PdumBase is highly expressed in early larvae.

SoxB and SoxC are important regulators of early neurogenesis. Still, the evolution and orthology relationships of *SoxB* genes in Lophotrochozoa and the relationship to vertebrate *SoxB1* and *SoxB2* is poorly understood [22, 24, 38]. This may be due to low taxon sampling in the respective phylogenetic analyses. Thus, we performed a broad taxon sampling, generated a first unrooted tree across most existing *Sox* genes, and found SoxC, SoxD, SoxE, SoxF, and at least a major part of *SoxB* well supported (Additional file 22). A second analysis focusing specifically on *SoxB* provides clear evidence that the *SoxB1* group predates Bilateria and that *Mfu-SoxB1* is orthologous to *SoxB1* from *C. teleta*, *P. dumerilii*, and *Crassostrea gigas* (Additional file 23). *Mfu-SoxB2* likewise falls in a well-supported group, which contains the vertebrate *SoxB2* representatives *Sox-21* and *Sox-14* and also *C. teleta SoxB* and *P. dumerilii SoxB*, which we, accordingly, regard as *SoxB2* orthologs. The basal branching of the *SoxB* group remains, however, elusive. We could not detect the expression of *Mfu-SoxB2* in the stages analyzed, but we got clear expression patterns for *Mfu-SoxB1* and *Mfu-SoxC*.

### Expression of several neural developmental genes starts early at the anterior and posterior pole and is highly dynamic on the cellular level

**Fig. 5:**
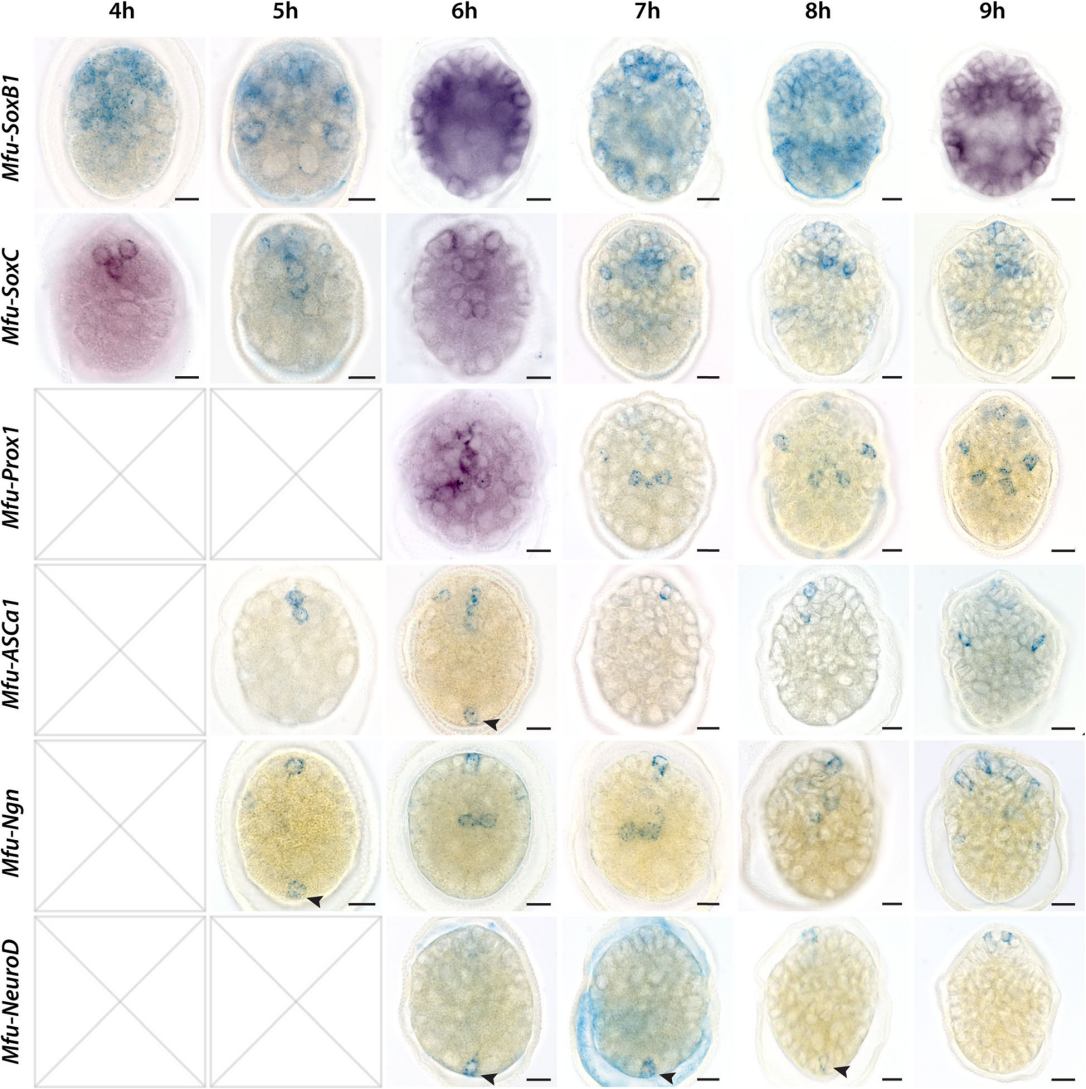
Expression of neural and proneural genes during 4–9 hpf stages of larval development. Scale bars: 20 μm

To determine the regions of neuronal specification during early development, we studied the expression of the aforementioned neural and bHLH class proneural genes. The bHLH class of proneural genes is known to be expressed in a transient manner in developing neurons [39]. Therefore, the expression of *bHLH* genes, but also *SoxB1*, *SoxC,* and *Prox1,* was investigated at 1-h intervals starting from 2 hpf stage. We could not detect any expression of the genes studied at 2hpf and 3hpf. The first gene being expressed is *Mfu-SoxB1*, which at 4 hpf is only confined to few cells in the animal pole (Fig. 5). At 5 hpf, the expression of *Mfu-SoxB1* extends throughout the animal pole cells while excluding the larger cells at the vegetal pole (Fig. 5). Shortly later, at 6 hpf stage, the expression of *Mfu-SoxB1* spans throughout the body, and from 7 hpf stage, it is observed more prominently in the vegetal region. Around 12 hpf stage onwards, the expression of *Mfu-SoxB1* gets more segregated, which may hint to the emergence of dedicated regions of proliferation and differentiation (Additional file 25). From 18 hpf, however, it remains mainly restricted to the anterior domain, and this pattern continues into later stages (Additional file 25).

*Mfu-SoxC* appears around 4 hpf in 2–3 cells near the animal pole. By 5 hpf stage, more cells start expressing *Mfu-SoxC* (Fig. 5). From 9 hpf stage onwards, some expression can be detected close to the posterior region, albeit transiently, but no expression was detected in the position of the PPN (Fig. 5). In general, expression of *Mfu-SoxC* remains mainly confined to the anterior and mid domains with expression in the trunk region starting later from 12 hpf stage (Fig. 5, Additional file 25). The expression of *Mfu-SoxC* in later stages (24 and 48 hpf) is similar to that of *Mfu-SoxB1*, as both are mostly restricted to the anterior domain (Additional file 25).

*Mfu-Prox1* is expressed from 6 hpf in few cells in the mid-region. In subsequent stages, the expression also continues to expand mainly in the anterior and mid domains. From 12 hpf, the expression of *Mfu-Prox1* extends along the trunk and posterior regions and is mainly detected in pairs of cells (Additional file 25).

The expression of proneural genes *Mfu-ASCa1* and *Mfu-Ngn* were detected from 5 hpf. While *Mfu-ASCa1* is expressed in two cells in the anterior region, *Mfu-Ngn* was detected in both the anterior and posterior poles (Fig. 5). At 6 hpf *Mfu-ASCa1* and now, *Mfu-NeuroD* also starts to express in the posterior region (Fig. 5). The posterior expression signal of all three genes corresponds to the position of PPN we observed by immunohistochemistry from 9 hpf onwards. Expression of both *Mfu-ASCa1* and *Mfu-Ngn* in the posterior cell is more transient than *Mfu-NeuroD*, whose expression was observed from 6 hpf stage until around 8 hpf stage (Fig. 5). From 9 hpf stage onwards, none of the proneural genes were detected in the posterior cell, whereas expression domains expand in the anterior region (Fig. 5, Additional file 26).

Other Achaete scute genes – *Mfu-ASCa2*, *Mfu-ASCa3*, and *Mfu-ASCa4* also begin expressing from 7 to 10 hpf stage onwards in an apparent non-overlapping manner (Additional file 26). The expression of *Mfu-OligoA* and *Mfu-OligoB* were detected from 9 to 10 hpf in a few cells in the anterior and along the developing trunk (Additional file 26). The *Mfu-Oligo* genes are expressed in restricted domains in comparison to other bHLH proneural genes. Around the stages 12–14 hpf, several proneural genes display an expanding pattern of expression (Additional file 26). In summary, the expression of proneural genes is dynamic, and many display broad patterns in later stages of development (Additional file 26).

### The first cell expressing proneural genes in the posterior region appears after 7 cleavages and is a descendant of the 2d blastomere

**Fig. 6:**
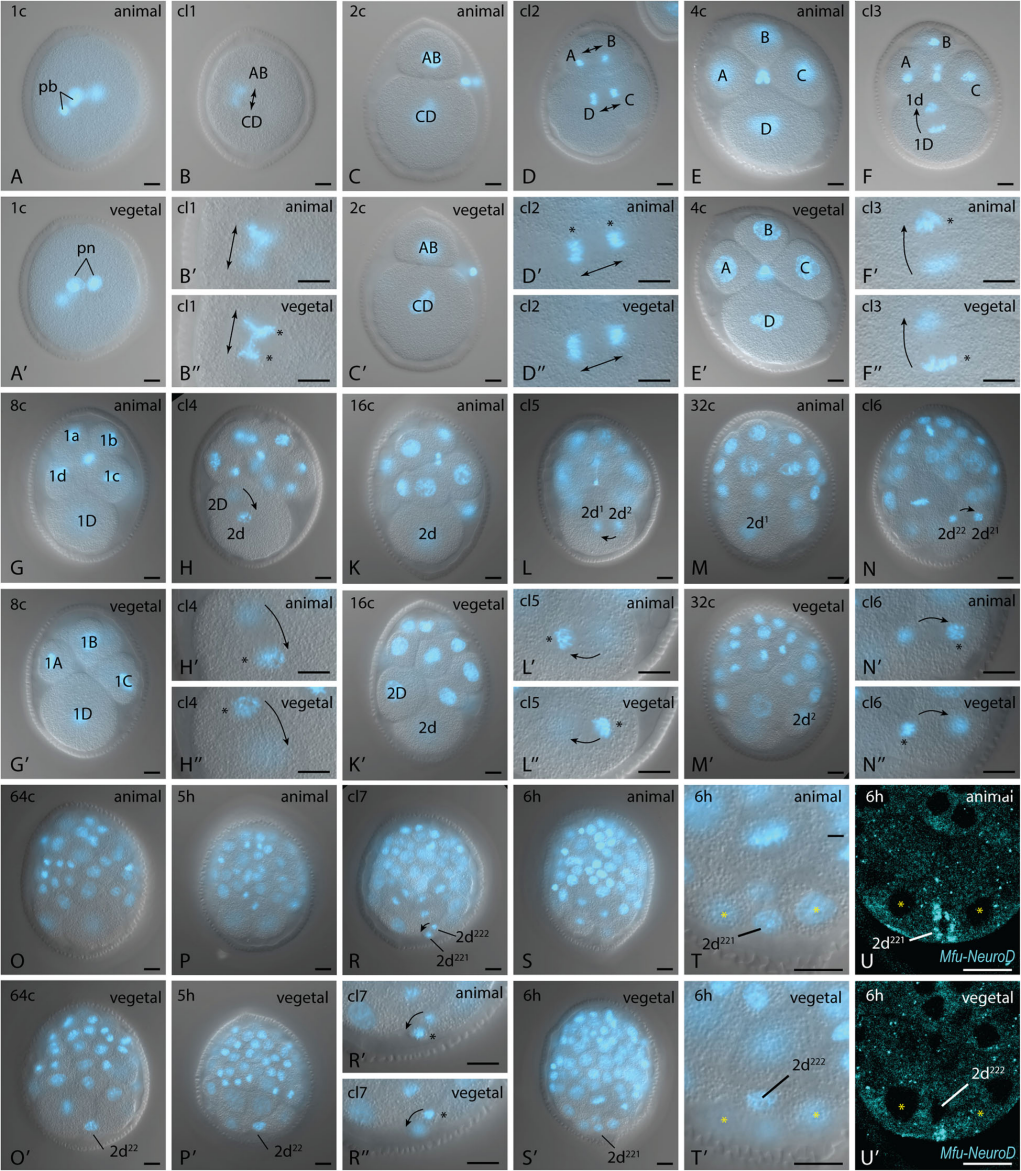
Clonal lineage of the cell 2d^221^ in the embryo of *M. fuliginosus*. **a**-**t’** Images of fixed, DAPI stained specimens from first to 7th cleavage. The images are labeled either by their stage (1c-64c, 5 h, 6 h) or by the cleavage (cl1-cl7). The positional labels animal and vegetal do not refer to the absolute position in the embryo, but to the relative position of image pairs of the same stage or cleavage. Arrows indicate the spindle direction. **u**-**u′** FISH with probe against *Mfu-NeuroD* at 6 h stage, posterior part of the embryo. (Yellow asterisks) corresponding landmarks between image pairs, (black asterisks) spindle pole in focus plane, (pb) polar body, (pn) pronucleus. Scale bars: 20 μm

Having detected the expression of proneural genes from 5 hpf onwards at the position where the PPN differentiates, we were interested in tracing the clonal origin of the cell at the posterior pole. For this purpose, we fixed embryos from 30 min to 6 h post-fertilization at every 10 min intervals. At least 100 specimens were used during each fixation. We then stained the nuclei with DAPI and recorded z-stacks of at least five larvae from each fixation (a total of 165 embryos). Since the pace of development differed to some extent between embryos, we ordered the image stacks up to the 64-cell stage not by time, but by the number of nuclei. We counted cleaving cells as two. At 6 hpf in the posterior pole, two small blastomeres are visible residing between two larger cells (Fig. 6s’, t, t’). We traced the posterior most of these cells back through development as being a descendant of the D quadrant. Daughter cells were named according to the spindle orientation and the relative position of the cells to each other along the animal-vegetal axis. Thirty minutes after fertilization, the zygote contains two polar bodies along with the male and female pronucleus (Fig. 1a, a’, Additional file 27). Twenty minutes later, the first cleavage generates a larger CD and a smaller AB cell (Fig. 6b, b’, b″, c, c′, Additional files 28, 29). The next cleavage gives rise to the 4-cell stage with a D-cell being considerably larger in size than the A, B, and C-cell (Fig. 6d, d’, d”, e, e’, Additional files 30, 31). The spindle orientation of the first two cleavages is perpendicular to the animal-vegetal axis and tilts upwards. The D-cell is the first cell that enters the third cleavage around 1 h 50 min post-fertilization is dextral and gives rise to a smaller animal 1d and a larger vegetal 1D-cell (Fig. 6f, f’, f″, g, g’, Additional files 32, 33). The next cleavage is sinistral and gives rise to the cells 2D and 2d (Fig. 6h, h’, h″, Additional file 34). Notably, in the 16-cell stage, the animal 2d cell is larger than the vegetal 2D cell (Fig. 6k, k’, Additional file 35). Cleavage 5 and 6 generate first the cell 2d^2^ on the right body side (Fig. 6l, l’, l”, 6m, m’, Additional files 36, 37), which then divides into 2d^21^ remaining on the right body side and 2d^22^ with a more central position (Fig. 6n, n’, n″, Additional file 38). The 2d^22^ travels further posterior until it reaches the posterior-most region in the 64-cell stage, which is around 5 hpf (Fig. 6o, o’, 6p, p′, Additional files 39, 40). Cleavage 7, finally, generates the two small blastomeres 2d^221^, which takes in the most posterior position in the embryo and 2d^222^, which lies a bit more vegetal and anterior (Fig. 6r, r’, r”, 6s, s′, Additional files 41, 42). At 6 hpf, the cells 2d^221^ and 2d^222^ are flanked left and right by two cells with large nucleus (Fig. 6t, t’). These nuclei are also useful landmarks after fluorescence in-situ hybridization. Between the large nuclei, the *Mfu-NeuroD* expression signal is surrounding a small nucleus, which corresponds to the position of 2d^221^ (Fig. 6u). Slightly anterior and vegetal is a second small nucleus that corresponds to the position of the cell 2d^222^ and is not surrounded by expression signal (Fig. 6u’).

## Discussion

Annelids, along with many other protostome invertebrates, share the presence of a brain and ventral longitudinal nerve cords. While homology of these parts of the CNS within annelids is widely accepted, it is still a matter of debate how the annelid trunk nervous system relates to that of other bilaterians. A highly similar mediolateral patterning of nerve cord development between annelids and distantly related bilaterians suggests a general homology [19, 20, 40]. The structural diversity of bilaterian nervous systems and molecular data on nervous system patterning from some groups were, however, also interpreted in favor of independent nerve cord evolution [41]. Molecular aspects of early neurogenesis have been studied in several species of Spiralia [21–24, 26, 42–44], but not on a cellular level and not with a focus on the pioneer neurons prefiguring the main scaffold of the later nervous system and on the dynamics of gene expression. Data on the first steps in nervous system formation are thus highly interesting from both a developmental and an evolutionary point of view.

### The general course of neurogenesis in *Malacoceros fuliginosus* – a variation of a common theme

The basic processes of neurogenesis, that is, specification of neurogenic areas, progression of progenitors, specification of precursors, and differentiation of neurons in *M. fuliginosus* largely follows a conserved pattern observed in many organisms. *SoxB1*, whose function in specification and proliferation of neural progenitors has been documented throughout the animal kingdom [45, 46] shows up very early in the prospective ectoderm and is expressed throughout the development of the nervous system in all of the investigated stages. *SoxC*, known to promote cell-cycle exit, follows a little later [47] is also amongst the early expressed genes. Then, several proneural genes of the bHLH families - *Neurogenin*, *NeuroD*, *ASC* and *Oligo* are broadly expressed, which are involved in neurogenesis in many organisms [1, 2, 44, 48]; and *Prox1* and *Elav*, typical markers of postmitotic precursors [49–51]; and finally markers for neural differentiation like *Synaptotagmin* and *Rab3*.

### The first steps in nervous system development in an annelid

The specific value of our dataset is that, for the first time, we can describe the development of an annelid nervous system in short time intervals — from the onset of the expression of a broad set of neural genes to the first signs of differentiation and the outgrowth of the first neurites which form the scaffold of the later nervous system. Further, we reveal the clonal origin of one of the most important cells, the pioneer of the ventral nerve cord. From these data, we get insights into the dynamics of neural gene expression partly on the cellular level. We can also link the expression of neural genes to the specificities of spiral cleavage, and the data may serve as a sound basis for experimental studies on nervous system development and comparative and evolutionary studies across species and groups.

We do not have evidence that any of the studied genes are maternally expressed in the early embryos when neurogenic regions and the fate of prospective neurons are specified. At 2 hpf and 3 hpf, we did not get any positive signal on the expression of *Sox*, *Prox1*, and any proneural bHLH gene. *SoxB1* and *SoxC* expression start at the animal pole around 4 hpf, where according to the embryological data, the vegetal pole is not yet covered by a layer of smaller blastomeres. The extension of *SoxB* and *SoxC* towards the vegetal pole during the next 2–3 h seems to correlate with epibolic coverage of the vegetal macromeres. Though, *SoxB* expression extends entirely to the vegetal pole or the posterior end; we could not observe the expression of *SoxC* in these regions.

Before *SoxB1* expression extends towards the vegetal pole, expression of some proneural genes already starts in the PPN. We detected the first expression of few bHLH genes at around 5 hpf, wherein the embryos have completed the 6th cleavage, and most cells are soon entering the 7th cleavage. During the early stages, the expression is restricted to only very few cells in the anterior and the posterior region, and this pattern remains until the first neurons differentiate at around 9 hpf. The expression of proneural genes becomes broader when the first neurons already express differentiation markers and send out the first neurites. The axons of few cells create the main routes of the central nervous system. The axon of an apical ganglion (AN1) travels on one body side down towards to the prototroch and probably further towards the prospective course of the VNC. From the other side, the single bifurcating PPN sends axons upwards towards the prototroch region. The prototroch ring nerve is formed by two pioneers (PNNs) on the ventral side of the prototroch. Mainly in the anterior hemisphere, soon more cells are developing, which mostly project towards the prospective brain region. At the posterior end, another neuron differentiates, which, as a follower, sends axons along the axons of the VNC pioneer. Early expression of neural genes in the anterior region may partly result from the transcriptional activity of the AN1. Still, we were not able to localize the expression patterns clearly in this cell since other cells also start developing early. The paired early expression signals of *Ngn* and *Prox1* in the middle of the body may likely be localized to the PNNs. Precise cellular localization of neural gene expression is possible in the PPN since we can observe it already at 9 hpf by immunohistochemistry and that we can see *NeuroD* expression up to 8 hpf in this specific position.

From the cellular localization pattern, we infer that the expression of some proneural genes is highly dynamic and differ in their regulation in different cells. While *Ngn* expression in the PPN is only at 5 hpf, it is likely to be expressed from 6 to 7 hpf in the PNNs, and 5–9 hpf in an anterior cell. ASCa1 is also expressed at one stage (6 hpf) in the PPN, while *NeuroD* remains expressed for 3 h. The expression of many proneural genes is known to be transient and is downregulated prior to the differentiation phase [39]. In *M. fuliginosus*, the expression of *Ngn*, *ASCa1*, and *NeuroD* genes in the PPN is transient, lasting only for one to 3 h, and ceases expressing before differentiation starts at around 9 hpf.

The inference of gene dependency or co-regulation of genes based on co-expression analyses is always speculative. The observed dynamics of gene expression provides an additional obstacle for this approach since the periods of overlapping expression in specific cells may be very brief. Nonetheless, it is worth noting that *Ngn* is the first proneural gene in the PPN, followed by *ASCa1* and *NeuroD*. Interestingly, *Ngn* expression in the posterior region is only at a time point when the 7th cleavage of the posterior pioneer precursor is not completed in all embryos of a batch. During vertebrate neurogenesis, *Ngn* is transiently expressed in proliferating precursors, which are then committed to a neural fate [52]. Similarly, expression of *Achaete-scute* and *Ngn* has been reported from proliferating neurons close to the ventral midline in later developmental stages in the annelid *Platynereis dumerilii* [40]*.*

Seemingly, in the prototroch pioneers, *Ngn* precedes the expression of *Prox1*, which is known to initiate the specification of neurons after they stopped cycling [50]. Although the molecular mechanisms by which proneural genes regulate neurogenesis and specify subtypes are being extensively studied, important questions remain as to what their main function is: general neurogenesis or subtype specification or both. Based on the short time intervals of our gene expression studies, we have the impression that many of the bHLH genes, especially the *ASCa* gene paralogs, are differentially expressed and probably involved in defining neural subtypes.

### Expression of sox and bHLH genes in annelids

A comparison of the general course of neurogenesis with other annelid representatives must take the orthology relationships of the genes investigated and the staging of the embryos into account. The gene expression patterns mainly reveal differences in the time course of neural development along the anterior-posterior axis. In contrast to *P. dumerilii* and *Capitella teleta,* neurogenesis progresses at a more similar speed in the anterior, the trunk, and the posterior in *M. fuliginosus.* The differentiation markers *Synaptotagmin* and *Elav* show up in the anterior region in *P. dumerilii* and *M. fuliginosus* around 14 hpf, and also the onset of *Prox1* expression may be similar, as it is already expressed in several cells in 12 hpf in *P. dumerilii* [53]. However, in the trunk of *P. dumerilii, SoxB* is most broadly expressed around 24 hpf and stays active until 55 hpf [24], while we find broadest expression in the trunk of *M. fuliginosus* around 14 hpf and downregulation already around 18 hpf. The significance of the comparison is limited since the *SoxB* investigated in *P. dumerilii* [24] clusters within SoxB2 in our analysis, and we obtained expression patterns only for *SoxB1*. Only for *C. teleta* expression data of both *SoxB1* and *SoxB2* exist [22]. As they show overlapping expression, they may have a redundant function similar to arthropods rather than an antagonistic function as in vertebrates [22]. In the trunk, *Elav* and *Synaptotagmin* are reported from 32 hpf onwards in *P. dumerilii* [19], while they show up already at 14–18 hpf in the trunk of *M. fuliginosus*. In *C. teleta*, *SoxB*, *Prox,* and several bHLH proneural genes show up much later in the trunk and the posterior than in the anterior [22].

The expression of several bHLH proneural genes is likewise not easy to compare between annelids, especially of Achaete-scute. According to our analysis, *Mfu-ASCa1* (which shows up first and whose expression was detected in the PPN) and *Mfu-ASCa2* are closely related to *P. dumerilii Achaete-scute1* and *Achaete-scute2* and *C. teleta Cte-Ash2*. While *P. dumerilii Achaete-scute2* is not yet an annotated sequence, *Cte-Ash2* was shown to have a broad expression in several tissues and not further studied [21].

Both *P. dumerilii*, *Achaete-scute1,* and *Achaete-scute2* are known to be involved in nervous system development, but their activity is only described from later stages. In the study from [23, 40] the earliest stage described is 24 hpf, when the VNC is populated with many neurons and in [27] during brain development at 48 hpf. Notably, during VNC development in *P. dumerilii*, *Achaete-scute1*, and *Neurogenin* are expressed in proliferating cells [40]. This may explain why these genes are expressed so early in *M. fuliginosus*. In *M. fuliginosus, Neurogenin* may be expressed before completion of the last cleavage of the PPN precursor. The remaining *ASCa* genes of *M. fuliginosus*, *Mfu-ASCa3,* and *Mfu-ASCa4* are closely related to *Cte-Ash1* and a transcript from *P. dumerilii*, which, according to PdumBase is highly expressed in early stages, but for which no cellular expression data exist. *Cte-Ash1* is expressed early in the anterior region in *C. teleta* during the formation of the brain and extends later also to the VNC and the posterior end [21, 22]. *Mfu-ASCa3* and *Mfu-ASCa4* are also expressed relatively early in the anterior region, but not as early as *Mfu-ASCa1* and also not in the PPN. Since the expression of *Mu-ASCa4* starts around 8 hpf and from *Mfu-ASCa3* around 10 hpf it is unlikely that they are involved in the early development of the first pioneers we observed in the anterior, midbody and posterior region. *Neurogenin* and *NeuroD* are also fairly early expressed in *C. teleta* [22], and even a transient early expression is reported in the pygidium, but it remains unclear in what kind of cells and how they develop further.

Our gene expression data, due to the short time intervals, gives a first insight into the dynamic nature of proneural genes in annelids. And probably relevant for further studies on the function of the genes at the cellular level. Co-expression studies have to take into account that several of the genes are quickly up- and downregulated.

### Position and role of pioneer neurons in Lophotrochozoa

On a comparative level, the role of pioneer neurons in lophotrochozoans and other Spiralia have been studied by immunohistochemical analysis of differentiated neurons. Similar to *M. fuliginosus,* the first appearing neurons in many organisms were found in the anterior and/or the posterior region of the embryos. The observed variability in position, neurotransmitter, and projection raised many questions, whether the formation of the nervous system in these animals progresses rather from anterior to posterior, vice versa, or from both sides and from central to the periphery or the other way round. A comparison of data is biased by the fact that many studies focus mainly on the small subset of serotonergic and FMRFamidergic neurons, while our data show that in *M. fuliginosus* the earliest differentiating pioneer neurons are devoid of these neurotransmitters. Moreover, many other neurons are already present before the first serotonergic and FMRFamidergic neurons are discernable. Nevertheless, evolutionary conservation of some patterns can be inferred. A posterior VNC pioneer probably has been present in the last common ancestor of the two major annelid subgroups - Errantia and Sedentaria [54]. A posterior bifurcating neuron highly similar to the cell we investigated in *M. fuliginosus* was described from the errant annelids, *Phyllodoce maculate* [55] and *P. dumerilii* [31, 56] and the sedentary annelid, *Pomatoceros lamarckii* [57]. In all four species, the respective cell is located on the very posterior tip of the developing larva, sends a bundle of cilia to the exterior, and two very long axons anteriorly along the future developing VNC. These similarities strongly suggest homology. Thorough electron microscopy-based characterization of neural circuitry in 3-day-old larvae of *P. dumerilii* revealed that this cell beside its pioneering role also takes in a sensory-motor function by directly synapsing to multiciliated cells of the prototroch [58]. We could trace the axons of the PPN to the level of the prototroch making a similar circuitry conceivable in *M. fuliginosus*. Neurotransmitter content of the PPN seems to vary in annelids. While it is serotonergic in *Phyllodoce maculate* [55] and *Pomatoceros lamarckii* [57], it contains serotonin and FMRFamide in *P. dumerilii* [56, 58] and none of these transmitters in *M. fuliginosus*, even though the expression of Synaptotagmin and Rab3 suggest the capacity of transmitter release. Accordingly, the presence of this cell may be overlooked, if studies on nervous system development rely mainly on stainings against few neurotransmitters like FMRFamide and 5HT and not on broad neuronal markers and also if time intervals between studied stages are too big to trace the outgrowth of the first neurites. The PPN is likely lost in *C. teleta,* where a close examination of neural development provides no hints on the presence of such a cell [32]*,* while this is difficult to judge for few other annelid trochophores, where less detailed data exist.

In the anterior region, we found two different kinds of pioneer neurons in *M. fuliginosus*. While the prototroch ring nerve is pioneered by axons of two peripheral sensory cells (PNNs), the nerves running posteriorly from the apical plexus associated with the apical organ is pioneered by neurites of a ganglion cell (AN1). This challenges the view that the CNS of annelid trochophores is generally prefigured only by peripheral sensory cells [29, 55, 56, 59]. Like the PPN, the early anterior pioneer neurons of *M. fuliginosus* cannot be identified by studying serotonin or FMRFamide immunoreactivity. In accordance, cell-lineage data from *P. dumerilii* show that several cholinergic neurons appear much earlier in the anterior region than the first serotonergic neurons and that the former already forms a considerable mesh of neurites at 30 hpf [53]. Data from *C. teleta* show that the first differentiating neurons in the apical region are not peripheral sensory cells, but are from within the future brain [32].

In conclusion, it is likely that in the ancestor of errant and sedentary annelids, the first neurons pioneering the scaffold of the later CNS arose at the anterior as well as the posterior pole. While in the posterior only peripheral sensory cells take in this function, in the anterior, probably both peripheral sensory cells and central ganglion cells are involved. This mode of neural development may be valid for annelids in general, though the situation in the few basal branching groups needs further investigation. The question in which direction CNS formation in annelids is progressing encompasses different aspects that are not necessarily linked to each other. For the differentiation of the vast majority of CNS neurons, existing data points towards an anterior-posterior progression. On the other side, the suggested ancestral presence of pioneers in both the anterior and the posterior regions means that scaffold formation of the CNS started from both sides. From which region the first neurites extend faster in extant representatives is a matter of the exact onset of neurite outgrowth from the respective poles. The timing may be strongly affected by even small heterochronic changes in development underlying the observable variation between species.

If further evidence arises that cell-autonomous specification drives the formation of the first differentiating neurons in the anterior as well as in the posterior region of annelid trochophores, scaffold formation of the whole annelid CNS may be under the strong influence of neurons specified by maternally inherited factors. No molecular data exist from lophotrochozoans or Spiralia other than annelids on the neurogenesis and specification of early CNS pioneer neurons. Yet, a large number of immunohistochemical studies suggest that the first neurites prefiguring the main routes of the nervous system likewise develops from a few early developing cells in mollusks, nemerteans, and lophophorates. Obtaining deep data on pioneer neuron specification, cell-lineage, and role in axonal pathfinding in spiral cleaving animals may thus be highly informative for a better understanding of nervous system development and evolution.

### Cell lineage, spiral cleavage, and cell-autonomous specification

In the large taxon of Lophotrochozoa and Spiralia, the molecular development of the neurons which pioneer the main routes of the developing nervous system has not been studied in detail. By correlating immunohistochemical and gene expression data, we could trace the development of the VNC establishing pioneer, PPN, from 5 hpf. By studying the embryology of *M. fuliginosus,* we could also reveal the clonal origin of the cell. The PPN is a derivate of the D quadrant of the spirally cleaving embryo. More specifically, it is the cell 2d^221^, which can also be identified on the molecular level by transiently expressing *Neurogenin*, *NeuroD*, and *ASCa1*. In Spiralia, descendants of the first micromere quartet (1a-1d) are known to contribute anterior neurons and the brain [60–62]. A contribution of 2d descendants to the nervous system has also been repeatedly reported, especially to the trunk nervous system. Either by labeling early blastomeres [61, 62] or by arresting cleavage and studying tissue-specific markers. With the latter approach, it was shown that in *P. dumerilii* 2d^2^, 2d^12^ and 2d^112^ and 2d^1122^ are expressing neural markers after days of arrested cleavage and that 2d^2^ and 2d^12^ take in a posterior position in the embryo [63]. Our data confirm the contribution of 2d descendants to the trunk nervous system. More specifically, 2d^22^ plays a central role in trunk nervous system formation from the very beginning. It will be interesting to investigate whether other cells that branched off earlier or other 2d descendants contribute to the formation of the VNC in *M. fuliginosus*. Interesting candidates are the descendants of the cell 2d^222^. We could not finally clarify whether the direct precursor 2d^22^ already starts expressing *Neurogenin* before it divides into 2d^222^ and 2d^221^.

In spirally cleaving animals like annelids, mollusks or flatworms, inherited cell-intrinsic properties are important for fate specification of early blastomeres [64–67], a process which often is called cell-autonomous specification. mRNA segregation during several rounds of asymmetric cell divisions is supposed to be the main mechanism for passing on maternal factors [68–71]. It probably is most relevant for specification of early differentiating cells before regulative mechanisms mediated by extrinsic factors take over during later developmental stages.

The direct influence of cell-intrinsic properties on nervous system development in Spiralia, however, is poorly understood. Notably, most evidence has recently been provided from annelids, where data points towards cell-autonomous specification of neurons in the anterior region of the embryo. Correlation of 3D cell-lineage data of the trochophore episphere with gene expression data in *P. dumerilii* revealed that many of the later appearing neurons with bilateral symmetry and similar features do not share corresponding lineages in left-right opposing quadrants suggesting a position related conditional specification [53]. In difference, early differentiating neurons of the apical organ do not originate from bilateral symmetrical clones. They do not express several transcription factors involved in head regionalization and therefore pointing towards cell-autonomous specification. In *C. teleta,* separation of the micromeres 1a-1d from the rest of the embryo in the 8-cell stage leads to head-only partial larva with cells expressing the neuronal marker *Elav* and seen as evidence for cell-autonomous specification of the respective neural fate [72]. Notably, the AN1 in the future apical organ pioneering the anterior part of the circumesophageal connective in *M. fuliginosus* also does not have a bilateral symmetrical counterpart, which might as well indicate cell-autonomous specification. Nevertheless, this remains speculative and needs further investigation.

According to our study, the PPN (VNC pioneer neuron) may be an excellent study subject for future investigations on the mode of specification of early neurons and the involvement of cell-intrinsic factors. A known cell-lineage, the few numbers of cleavages, easy identification, and experimentally accessibility on the very posterior end may allow the application of a broad set of experimental tools. Respective studies, deep molecular characterization of the early pioneers, and molecular data on axon finding may deepen the understanding of nervous system development in Spiralia, and comparative studies may undoubtedly be interesting in the context of the ongoing debate on the homology of nerve cords in Spiralia and Bilateria.

## Conclusions

An anterior and posterior origin of the nervous system is likely ancestral for the vast majority of annelids due to the similarities we observed in the course of neural development in *Malacoceros fuliginosus* to that described in errant polychaetes. For the first time, we describe in an annelid in short time intervals the development of the first neurons appearing by the expression of Sox, proneural bHLH genes, and few other markers, the onset of neural differentiation, and the outgrowth of the first neurites, which create the primary neuronal scaffold. In *M. fuliginosus* both peripheral and ganglionic cells are amongst the first neurons contributing to the scaffold. Most of the early pioneers do not express serotonin and FMRFamide, which are commonly used as immunohistochemical markers for neuroanatomical studies. Expression of bHLH genes is transient in the first neurons and partly lasts only around 1 h suggesting very dynamic regulation. Brain precursors are known to arise from the 1a and 1c micromeres in spirally cleaving animals. Yet, a contribution of the blastomere 2d to the annelid and mollusc trunk nervous system has also been already claimed. We could in *M. fuliginosus* trace the lineage of the posterior pioneer neuron of the ventral nerve cord and show that it is the cell 2d^221^. Already after seven or even only six cleavages, this cell starts expressing proneural bHLH genes, which at this time also show up in the anterior. Cell-intrinsic factors play important roles in the early development in spirally cleaving animals. To which degree this also applies to the development of the nervous system is poorly studied, but the early onset of neural specification and differentiation of the early pioneers points into this direction. The cells we describe are probably promising study subjects for respective functional studies. Obtaining comparative data on the pioneers and their role in nervous system development across other groups with spiral cleavage will potentially contribute valuable insights for the ongoing debate on the homology of nerve cords in protostomes and Bilateria.

## Methods

### *Malacoceros fuliginosus* culture

Adult *M. fuliginosus* were collected from Pointe de Mousterlin, Fouesnant, France, and were maintained in sediment containing seawater tanks at 18 °C. Individual males and females were picked, rinsed several times with filtered seawater, and kept in separate bowls until they spawned. The staging was started from the time gametes were combined in a fresh bowl. Bowls were kept at 18 °C under 12:12 h light-dark cycle, and water was replaced every day or every second day. Larvae were fed with microalga *Chaetoceros calcitrans* from 24 hpf onwards after each water change.

### RNA-Seq and transcriptome assembly

Total RNA was extracted from cryofixed samples of various larval stages using the Agencourt RNAdvance Tissue Kit (Beckman Coulter). Library preparation and sequencing was performed by EMBL Genomics Core Facility (Heidelberg, Germany) using cation-based chemical fragmentation of RNA, Illumina Truseq RNA-Sample Preparation Kit and 1 lane of 100 bp paired end read sequencing on Illumina HiSeq 2000. Raw reads were trimmed, and error corrected with Cutadapt 1.2.1, the ErrorCorrectReads tool implemented in Allpaths-LG, and assembled with Trinity. A second assembly, including several steps for correction of sequence errors, chimerism, and elimination of redundancy, was generated using the DRAP pipeline [73] and the assemblers Trinity, rnaSPAdes, and Trans-ABySS, followed by gene clustering using Corset [74].

### Transcriptome screening and gene orthology

The transcriptomic resources of *M. fuliginosus* were deeply screened by similarity searches using tblastn for genes of interest. For bHLH group A genes, we used all Acheate-Scute (ASC), Atonal, Neurogenin, NeuroD, and Oligo amino acid sequences from *Capitella teleta* and *Crassostrea gigas* discovered by [36] as queries. We added the candidates which gave bHLH genes as hits by reciprocal blast against Genbank with MAFFT (option add) to the bHLH group A part of the bHLH gene alignment of [36]. In addition to ASC, Atonal, Neurogenin, NeuroD, and Oligo, the resulting alignment contains many other bHLH group A sequences. We computed an unrooted gene tree with IQ-TREE 1.5.5 with the model calculated by the included ModelFinder, SH-like approximate likelihood ratio test (1000 replicates), ultrafast bootstrap (1000 replicates) and approximate Bayes test for estimating branch support. We set unsuccessful iterations to stop tree searching to 500 and perturbation 380 strength to 0.2. The same parameters were used for all other gene tree inferences as well. To get closer insights into the evolution of the *Acheate-Scute (ASC)* family we aligned (MAFFT) all sequences, which clustered in the respective group in the tree inferred above and used the conserved region of the alignment as query profile for HMMER searches against Metazoa RP15 and against Annelida, Mollusca, Platyhelminthes, and Brachiopoda Uniprot with a cutoff-evalue of 1e-15. To get a better sampling in Annelida we also screened the Genbank annelid protein and the TSA database of *Platynereis dumerilii* by blast and tblastn (e-value cutoff 1e-10) with all sequences from *C. gigas* and *Capitella teleta* which clustered in the *bHLH* gene tree in the *ASC* clade. For those ASC sequences from *C. teleta* and *Nematostella vectensis,* which matched sequences used by [21, 75], we adopted the respective sequence annotations. We added all sequences from *M. fuliginosus,* which clustered in the ASC clade in the *bHLH group A* analysis above to the dataset. We removed duplicates and aligned the sequences with MAFFT (EINSI option) and kept only the conserved regions by manual curation. We removed sequences shorter than 60% of the alignment and calculated a tree with IQ-TREE with the same parameters as above. For POU genes, we screened the *M. fuliginosus* transcriptome resources with tblastn (e-value cutoff 1e-30) and all POU gene sequences from *C. teleta* uncovered by [76] as queries. We checked all hits for the presence of the POU domain with SMART. For a first analysis we added the retrieved sequences to the POU gene sampling of [76], aligned them with MAFFT (EINSI option), and inferred a gene tree with IQ-TREE with the same parameters as above. For a second analysis, we removed three sequences with the longest branches. Further, we screened the Genbank protein database with all POU genes found in *M. fuliginosus* (blastp, e-value cutoff 1e-80). We kept only the longest isoforms, aligned the sequences with MAFFT (option EINS), and kept only the well-conserved regions containing the POU domain and the homeodomain. The gene tree was inferred as above. For Elav and CUGBP Elav-like, we generated a query profile by aligning (MAFFT EINSI) sequences from Mouse, *Drosophila melanogaster*, *Tribolium castaneum,* and few Mollusk and annelid species and kept only conserved regions. With this profile, we conducted HMMER searches against RefSeq Metazoa (e-value cutoff 1e-100). We added few sequences with annotation nucleolysin TIA-1 and an e-value of 1E-39 as a potential outgroup. We removed highly similar sequences by clustering with 3 runs of h-cd-hit with identity cutoffs of 0.9, 0.7 and 0.6. For screening the *M. fuliginosus* transcriptome ressources we used several Elav and CUGBP elav-like sequences as queries. The candidates retrieved were than used as queries against Genbank Lophotrochozoa (first 100 hits (only longest isoforms)). We removed duplicates from the data set, aligned it with MAFFT (EINSI option) and kept only the conserved regions by manual curation. We computed the gene with IQ-TREE as above. To identify *prospero1 (Prox1)* orthologs, we used the PFAM PF05044 profile alignment of the prospero domain as query profile for HMMER searches against RP15 (e-value cutoff 1E-10). From the resulting dataset we used the sequences from *C. teleta*, *Helobdella robusta*, *C. gigas* and *Mizuhopecten yessoensis* as queries for similarity searches with tblastn and blastp against the *M. fuliginosus* transcriptome and Genbank Lophotrochozoa proteins (e-value cutoff 1e-10 in both cases). *Prox1* is a fairly derived group of genes. Thus, to prevent impact of non-homologous sites on the alignment of conserved regions of the Prox1 sequences, we did not add outgroup sequences to the sampling. We removed duplicates, kept only the longest isoforms, aligned the sequences with MAFFT (EINSI) and kept only well conserved regions by manual curation. The tree was inferred with IQ-TREE with the same parameters as above. To identify *SoxB* and *SoxC* orthologs, we used the alignment of conserved Sox gene domains from [77] and [78] as query profiles for HMMER searches against Uniprot Annelida, Mollusca, Brachiopoda and Platyhelminthes and Metazoa RP15 with e-value cutoff of 1e-30. We kept a copy of all sequences of *Homo sapiens*, *Mus musculus*, *Danio rerio*, *Strongylocentrotus purpuratus*, *D. melanogaster*, *Anopheles gambia*, *T. castaneum*, *Caenorhabditis elegans*, *C. teleta*, *P. dumerilii*, *C. gigas*, *Lottia gigantea*, *Schmidtea mediterranea*, and *N. vectensis* and clustered the sequences clustered with h-cd-hit (with identity cutoffs of 0.9, 0.6 and 0.3) to reduce the number of sequences. We then added the separately kept sequences and removed duplicates. We screened the transcriptome ressources of *M. fuliginosus* by tblastn with all sequences retrieved above from *C. teleta* and *C. gigas* as queries and an e-value cutoff of 1e-30. We removed the mammalian sex determination SRY genes from the sampling as they are fairly diverged. For a first analysis with the main aim to identify SoxB and SoxC sequences of *M. fuliginosus* we aligned the sequenes with MAFFT (option LINSI), kept only the conserved residues in the the regions of the HMG box domain and calculated an unrooted tree with IQ-TREE (same parameters as above). For getting closer insights into *SoxB* evolution we used all sequences, which clustered in the above analysis with known SoxB sequences and few SoxC and SoxD sequences for a second analysis. We aligned the SoxB sequences again with MAFFT (option LINSI) and edited the alignment less restrictively. Especially downstream of the HMG box, we kept more residues. We added the SoxC and SoxD sequenes to this alignment with MAFFT (option add) and calculated a tree with IQ-TREE (same parameters as above) and rooted it with SoxD. We checked the *M. fuliginosus* SoxB sequences for presence of the HMG domain with the SMART domain and InterproScan web servers.

### Gene cloning and probe generation

Specific primers were designed for genes of interest retrieved from the transcriptome resources of *M. fuliginosus,* and fragments were amplified from either mixed stage or stage-specific cDNA. The PCR fragments were inserted into pGEM-T-easy vector (Promega) and cloned in Top10 *E. coli* cells (Invitrogen). DIG/FITC labeled RNA probes were generated by DIG RNA labeling mix (Roche) or using transcription reagents along with DIG-UTP/FITC-UTP (Roche) by SP6 or T7 polymerases (Roche).

### In-situ hybridization

All animals were fixed in 4% PFA (in 1X PBS, 0.1% Tween20) for 2.5 h. Larvae 48 h and older were first relaxed with 1:1 MgCl_2_-seawater for 3–5 min before fixing them in 4% PFA (in 1X PBS, 0.1%Tween20) for 2.5 h. Samples were stored at − 20 °C in methanol until use. The larvae were rehydrated in series of 75, 50, and 25% methanol in PTW (1X PBS pH 7.4, 0.1% Tween20). Tissue was permeabilized by Proteinase K (100 ng/ml) treatment (30 s for 24 h larvae to 3 min for 5 d larvae). This was followed by two 5 min Glycine washes (2 mg/ml). The larvae were then acetylated with 1% triethanolamine (TEA) in PTW for 5 min and 0.5 μl/ml acetic acid in 1% TEA for 5 min. Two 5 min washes with PTW were done before fixing the larvae for 15 min in 4% PFA (in 1X PBS-Tween20). The post-fixed samples were washed four times in PTW for 5 min each. The larvae were then equilibrated in hybridization solution (Hyb solution: 50% formamide, 5X SSC, 50 μg/ml heparin, 100 μg/ml salmon sperm DNA, 0.1% Tween20, 1% SDS and 5% dextran sulphate (Alfa Aesar J63690, MW 40,000) in sterile water) for 10 min. Prehybridization was performed with Hyb solution at 65 °C for 2–4 h before hybridization with labeled RNA probes at a concentration of 1 ng/μl to 2.5 ng/μl for 48–60 h at 65 °C. After this, the samples were subjected to two post-hybridization washes of 5 min and 20 min with Hyb solution at 65 °C. The next washes with SSC were done as following – 2X SSC-Hyb solution in series of 25, 50, 75, and 100% 2X SSC each for 10 min at 65 °C, followed by two 30 min 0.05X SSC washes at 65 °C. The samples were then kept at RT for 10 min before washing in PTW-0.05X SSC in series of 25, 50, 75, and 100% PTW for 5 min each. Blocking was done with Roche Blocking solution (in Maleic acid buffer pH 7.5) for 1 h and then incubated in anti-DIG-AP or anti-FITC-AP (Roche) Fab fragments (1:5000) overnight at 4 °C. After this, the larvae were washed six times in PTW for 1 h and then equilibrated first in Mg_2_-free AP buffer (two 5 min washes), then in AP buffer (100 mM NaCl, 50 mM MgCl_2_, 100 mM Tris (pH 9.5 for NBT/BCIP staining and pH 8.2 for Fast Blue/Fast Red staining), 0.1% Tween20) (two 5 min washes). Probe detection was performed using NBT/BCIP (Roche) in AP buffer (pH 9.5) by adding 2.25 μl/ml NBT (from 100 mg/ml stock) and 3.5 μl/ml BCIP (from 50 mg/ml stock). Fast Blue (Sigma)/Fast Red (Roche) single or double staining was according to the protocol described in [79].

### Immunolabeling

All animals were fixed in 4% PFA (in 1X PBS, 0.1% Tween20) for 30 min. Larvae 48 h and older were first relaxed with 1:1 MgCl_2_-seawater for 3–5 min before fixing them in 4% PFA (in 1X PBS, 0.1% Tween20) for 30 min. After fixation, the samples were washed two times in PTW, followed by two washes in THT (0.1 M Tris pH 8.5, 0.1% Tween20). Blocking was in 5% sheep serum in THT for 1 h before incubating in primary antibodies (Monoclonal anti-acetylated α-tubulin, 1:300 Sigma product number T6793; Anti-5-HT, 1:500, Immunostar product number 20080; Anti-FMRFamide, 1:500, Immunostar product number 20091) for 48 h at 4 °C. The samples were then subjected to two 10 min washes in 1 M NaCl in THT followed by five 30 min washes in THT before incubating in secondary antibodies (Alexa Fluor 1:500, Thermo Fisher Scientific) overnight at 4 °C. Next, the samples were washed in THT, two 5 min washes followed by five 30 min washes. Specimens were stored in embedding medium (90% glycerol, 1x PBS, and 2% DABCO) at 4 °C.

### Embryology

For studying embryology, we fixed larvae from a single batch starting from 30 mpf up to 6 hpf at every 10 min intervals. We used at least 100 larvae for each fixation (1 h in 4% PFA). Next, the larvae were rinsed several times with PTW, stained with DAPI for 1 h, and rinsed again with PTW. The stained specimens were then transferred to the mounting medium (Glycerol with 2% DABCO). We studied at least 5 embryos from each fixation and recorded two-channel (DIC and DAPI) Z stacks with a Zeiss Axio Imager Z2. In total, 165 embryos were studied.

### Microscopy and image processing

Samples were mounted in glycerol and imaged using the Zeiss Examiner A1 microscope. Confocal imaging was done with a Leica SP5 microscope. Images were processed using ImageJ, Adobe Photoshop CS6, Imaris (Bitplane), and assembled using Adobe Illustrator CS6.

## Supplementary information

**Additional file 1.** Nervous system at 9 hpf. Movie file (.avi). CLSM image stack of whole larva (dorsal to ventral). Staining against acetylated alpha-tubulin (green) and nuclei (DAPI, blue).

**Additional file 2.** Nervous system at 14 hpf. Movie file (.avi). CLSM image stack of whole larva (dorsal to ventral). Staining against acetylated alpha-tubulin (green), FMRFamide (magenta) and nuclei (DAPI, blue).

**Additional file 3.** Nervous system at 14 hpf. Movie file (.avi). CLSM image stack of whole larva (ventral to dorsal). Staining against acetylated alpha-tubulin (green), FMRFamide (magenta) and nuclei (DAPI, blue).

**Additional file 4.** Posterior pioneer neuron at 14 hpf. Movie file (.avi). CLSM image stack of the posterior region (dorsal to ventral). Staining against acetylated alpha-tubulin (green) and nuclei (DAPI, blue).

**Additional file 5.** Nervous system at 16 hpf. Movie file (.avi). CLSM image stack of whole larva (ventral to dorsal). Staining against acetylated alpha-tubulin (green), FMRFamide (magenta) and nuclei (DAPI, blue).

**Additional file 6.** Nervous system at 16 hpf. Movie file (.avi). CLSM image stack of whole larva (dorsal to ventral). Staining against acetylated alpha-tubulin (green), FMRFamide (magenta) and nuclei (DAPI, blue).

**Additional file 7.** Posterior pioneer neuron at 16 hpf. Movie file (.avi). CLSM image stack of the posterior region (ventral to dorsal). Staining against acetylated alpha-tubulin (green) and nuclei (DAPI, blue).

**Additional file 8.** Nervous system at 19 hpf. Movie file (.avi). CLSM image stack of whole larva (dorsal to ventral). Staining against acetylated alpha-tubulin (green), 5-HT (magenta) and nuclei (DAPI, blue).

**Additional file 9.** Nervous system at 19 hpf. Movie file (.avi). CLSM image stack of whole larva (dorsal to ventral). Staining against acetylated alpha-tubulin (green), FMRFamide (magenta) and nuclei (DAPI, blue).

**Additional file 10.** Nervous system at 21 hpf. Movie file (.avi). CLSM image stack of whole larva (dorsal to ventral). Staining against acetylated alpha-tubulin (green), 5-HT (magenta) and nuclei (DAPI, blue).

**Additional file 11.** Nervous system at 21 hpf. Movie file (.avi). CLSM image stack of whole larva (ventral to dorsal). Staining against acetylated alpha-tubulin (green), FMRFamide (magenta) and nuclei (DAPI, blue).

**Additional file 12.** Posterior pioneer neuron at 21 hpf. Movie file (.avi). CLSM image stack of the posterior region (dorsal to ventral). Staining against acetylated alpha-tubulin (green) and nuclei (DAPI, blue).

**Additional file 13.** Nervous system at 24 hpf. Movie file (.avi). CLSM image stack of whole larva (ventral to dorsal). Staining against acetylated alpha-tubulin (green) and nuclei (DAPI, blue).

**Additional file 14.** Nervous system at 24 hpf. Movie file (.avi). CLSM image stack of whole larva (ventral to dorsal). Staining against acetylated alpha-tubulin (green), FMRFamide (magenta) and nuclei (DAPI, blue).

**Additional file 15.** Nervous system at 24 hpf. Movie file (.avi). CLSM image stack of the anterior region larva (dorsal to ventral). Staining against acetylated alpha-tubulin (green), 5-HT (magenta) and nuclei (DAPI, blue).

**Additional file 16.** Nervous system at 24 hpf. Movie file (.avi). CLSM image stack of the anterior region larva (dorsal to ventral). Staining against acetylated alpha-tubulin (green), FMRFamide (magenta) and nuclei (DAPI, blue).

**Additional file 17 **Evolution of Elav and CUGBP-Elav-like genes. Adobe Acrobat file (.pdf). Maximum-likelihood tree (IQ-TREE, model LG + R5 chosen by Modelfinder). Branches with approximate Bayes test ≥0.98 are labelled. Sequences of *M. fuliginosus* are highlighted in red. Genes we found being expressed in the analyzed stages are marked by arrows.

**Additional file 18 **Evolution of POU genes . Adobe Acrobat file (.pdf). Maximum-likelihood tree (IQ-TREE, model LG + R6 chosen by Modelfinder). Branches with approximate Bayes test ≥0.98 are labelled. Sequences of *M. fuliginosus* are highlighted in red. Genes we found being expressed in the analyzed stages are marked by arrows.

**Additional file 19 **Evolution of Prox1 genes. Adobe Acrobat file (.pdf). Unrooted maximum-likelihood tree (IQ-TREE, model LG + R4 chosen by Modelfinder). Branches with approximate Bayes test ≥0.98 are labelled. Sequences of *M. fuliginosus* are highlighted in red. Genes we found being expressed in the analyzed stages are marked by arrows.

**Additional file 20 **Evolution of bHLH group A genes. Adobe Acrobat file (.pdf). Unrooted maximum-likelihood tree (IQ-TREE, model LG + R6 chosen by Modelfinder). Branches with approximate Bayes test ≥0.98 are labelled. Sequences of *M. fuliginosus* are highlighted in red. Genes we found being expressed in the analyzed stages are marked by arrows.

**Additional file 21 **Evolution of achaete scute (ASC) genes. Adobe Acrobat file (.pdf). The tree is based on a subset of the analysis shown in Fig. S4 with an ASC-specific and longer alignment. Maximum-likelihood tree (IQ-TREE, model JTTDCMut+R6 chosen by Modelfinder). Branches with approximate Bayes test ≥0.98 are labelled. Sequences of *Malacoceros fuliginosus* are highlighted in red, sequences of *Capitella teleta* in green, of *Platynereis dumerilii* in blue and of *Nematostella vectensis* in yellow. Genes we found being expressed in the analyzed stages are marked by red arrows. Genes of *C. teleta*, *P. dumerilii,* and *N. vectensis*, which are reported to be expressed in the context of nervous system development are also marked by arrows. Dashed arrows are used for sequences which are also broadly expressed in other tissues. For genes of *P. dumerilii* the expression level is indicated based on data in PdumBase.

**Additional file 22 **Evolution of Sox genes. Adobe Acrobat file (.pdf). Unrooted maximum-likelihood tree (IQ-TREE, model LG + R8 chosen by Modelfinder). Branches with approximate Bayes test ≥0.98 are labelled. Sequences of *M. fuliginosus* are highlighted in red. Genes we found being expressed in the analyzed stages are marked by arrows.

**Additional file 23 **Evolution of SoxB genes. Adobe Acrobat file (.pdf). The tree is based on a subset of the analysis shown in Fig. S6 with a SoxB-specific sequence and longer alignment. Maximum-likelihood tree (IQ-TREE, model JTT + R5 chosen by Modelfinder). Branches with approximate Bayes test ≥0.98 are labelled. Sequences of *M. fuliginosus* are highlighted in red. Genes we found being expressed in the analyzed stages are marked by arrows.

**Additional file 24 **Expression of synaptic genes *Mfu-Syt1* and *Mfu-Rab3* in stages 48–72 hpf. Image file (*.tif). (A,B) WMISH of *Mfu-Syt1* and *Mfu-Rab3* at 48 hpf. (C) FISH of *Mfu-Syt1* at 72 hpf showing the expression in the VNC. Scale bars: 50 μm.

**Additional file 25 **Expression of *Mfu-SoxB1*, *Mfu-SoxC,* and *Mfu-Prox1* from 12 hpf onwards. Image file (*.tif). (A-L) WMISH in stages 12–24 hpf. (M) Double ISH of *Mfu-SoxB1* and *Mfu-SoxC* at 48 hpf*.* Scale bars: 20 μm.

**Additional file 26.** Expression of proneural bHLH genes in stages 7–14 hpf. Image file (*.tif). Scale bars: 20 μm.

**Additional file 27.** 1-cell stage at 30 min post fertilization. Movie file (*.avi). Fixed specimen stained with DAPI. Image stack from animal to vegetal pole recorded with DIC and DAPI channel (blue).

**Additional file 28.** Cleavage 1 at 50 min post fertilization. Movie file (*.avi). Fixed specimen stained with DAPI. Image stack from animal to vegetal pole recorded with DIC and DAPI channel (blue).

**Additional file 29.** 2-cell stage at 50 min post fertilization. Movie file (*.avi). Fixed specimen stained with DAPI. Image stack from animal to vegetal pole recorded with DIC and DAPI channel (blue).

**Additional file 30.** Cleavage 2 at 1 h 40 min post fertilization. Movie file (*.avi). Fixed specimen stained with DAPI. Image stack from animal to vegetal pole recorded with DIC and DAPI channel (blue).

**Additional file 31.** 4-cell stage at 2 h post fertilization. Movie file (*.avi). Fixed specimen stained with DAPI. Image stack from animal to vegetal pole (specimen is slightly tilted due to the size differences between macromeres) (specimen is slightly tilted due to the size differences between macromeres) recorded with DIC and DAPI channel (blue).

**Additional file 32.** Cleavage 3 of D-cell at 1 h 50 min post fertilization. Movie file (*.avi). Fixed specimen stained with DAPI. Image stack from animal to vegetal pole (specimen is slightly tilted due to the size differences between macromeres) recorded with DIC and DAPI channel (blue).

**Additional file 33.** 8-cell stage at 2 h 30 min post fertilization. Movie file (*.avi). Fixed specimen stained with DAPI. Image stack from animal to vegetal pole (specimen is slightly tilted due to the size differences between macromeres) recorded with DIC and DAPI channel (blue).

**Additional file 34.** Cleavage 4 of 1D-cell at 2 h 20 min post fertilization. Movie file (*.avi). Fixed specimen stained with DAPI. Image stack from animal to vegetal pole (specimen is slightly tilted due to the size differences between macromeres) recorded with DIC and DAPI channel (blue).

**Additional file 35.** 16-cell stage at 2 h 40 min post fertilization. Movie file (*.avi). Fixed specimen stained with DAPI. Image stack from animal to vegetal pole (specimen is slightly tilted due to the size differences between macromeres) recorded with DIC and DAPI channel (blue).

**Additional file 36.** Cleavage 5 of 2d-cell at 3 h 10 min post fertilization. Movie file (*.avi). Fixed specimen stained with DAPI. Image stack from animal to vegetal pole (specimen is slightly tilted due to the size differences between macromeres) recorded with DIC and DAPI channel (blue).

**Additional file 37.** 32-cell stage at 3 h 20 min post fertilization. Movie file (*.avi). Fixed specimen stained with DAPI. Image stack from animal to vegetal pole (specimen is slightly tilted due to the size differences between macromeres) recorded with DIC and DAPI channel (blue).

**Additional file 38.** Cleavage 6 of of 2d^2^-cell at 3 h 30 min post fertilization. Movie file (*.avi). Fixed specimen stained with DAPI. Image stack from animal to vegetal pole (specimen is slightly tilted due to the size differences between macromeres) recorded with DIC and DAPI channel (blue).

**Additional file 39.** 64-cell stage at 4 h 20 min post fertilization. Movie file (*.avi). Fixed specimen stained with DAPI. Image stack from animal to vegetal pole (specimen is slightly tilted due to the size differences between macromeres) recorded with DIC and DAPI channel (blue).

**Additional file 40.** 5 h post fertilization stage. Movie file (*.avi). 5hpf stage. Fixed specimen stained with DAPI. Image stack from animal to vegetal pole (specimen is slightly tilted due to the size differences between macromeres) recorded with DIC and DAPI channel (blue).

**Additional file 41.** Cleavage 7 of 2d^22^-cell at 5 h 20 min post fertilization. Movie file (*.avi). Fixed specimen stained with DAPI. Image stack from animal to vegetal pole (specimen is slightly tilted due to the size differences between macromeres) recorded with DIC and DAPI channel (blue).

**Additional file 42.** 6 h post fertilization stage. Movie file (*.avi). Fixed specimen stained with DAPI. Image stack from animal to vegetal pole (specimen is slightly tilted due to the size differences between macromeres) recorded with DIC and DAPI channel (blue).

## Data Availability

Sequence data are available in GenBank (Accession numbers MT901642 - MT901672).

## Abbreviations

AN1: Apical neuron 1
CNS: Central nervous system
PNN: Prototroch nerve forming neuron
PPN: Posterior pioneer neuron
VNC: Ventral nerve cord
