# Supplementary figures and images for: The development of early pioneer neurons in the annelid Malacoceros fuliginosus
Source: BMC Evol Biol. 2020 Sep 14;20:117. doi: 10.1186/s12862-020-01680-x (PMC7489019; doi:10.1186/s12862-020-01680-x)

Tree scale: 0.1

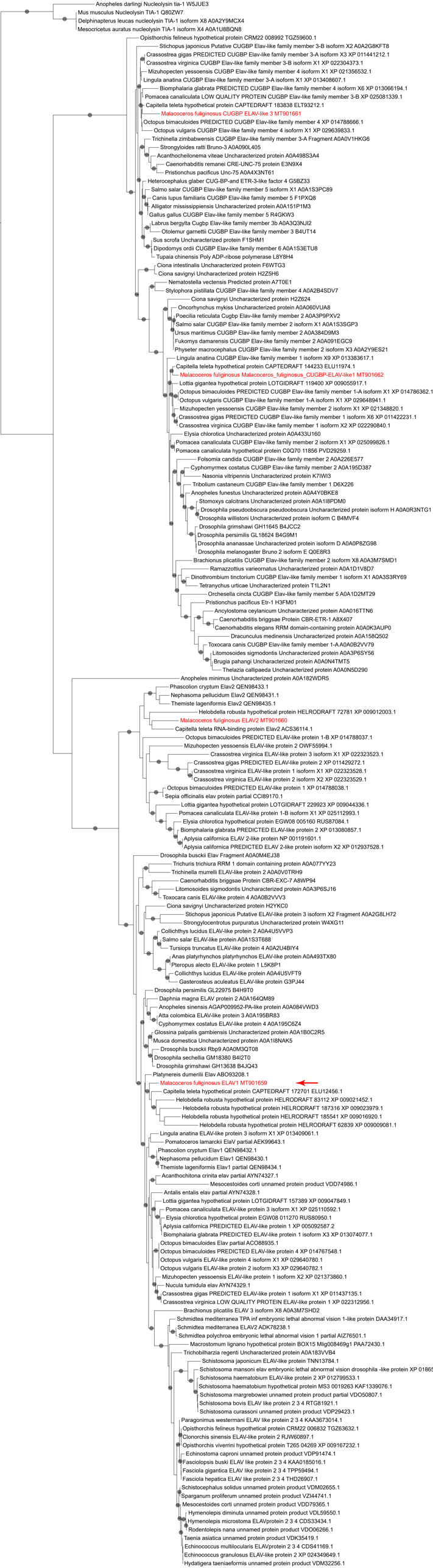

CUGBP ELAV-like

ELAV2

ELAV

ELAV1

Supplement: Supplementary file 17 — Additional file 17 Evolution of Elav and CUGBP-Elav-like genes. Adobe Acrobat file (.pdf). Maximum-likelihood tree (IQ-TREE, model LG + R5 chosen by Modelfinder). Branches with approximate Bayes test ≥0.98 are labelled. Sequences of M. fuliginosus are highlighted in red. Genes we found being expressed in the analyzed stages are marked by arrows. [file 12862_2020_1680_MOESM17_ESM.pdf]

Tree scale: 0 1 

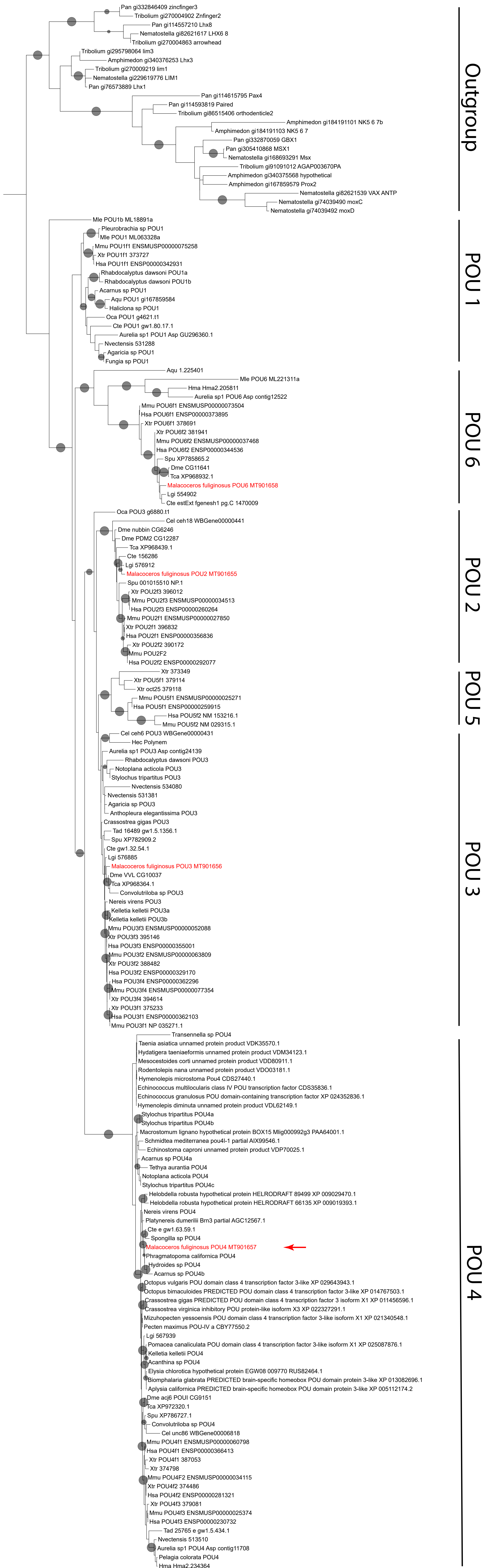

Supplement: Supplementary file 18 — Additional file 18 Evolution of POU genes . Adobe Acrobat file (.pdf). Maximum-likelihood tree (IQ-TREE, model LG + R6 chosen by Modelfinder). Branches with approximate Bayes test ≥0.98 are labelled. Sequences of M. fuliginosus are highlighted in red. Genes we found being expressed in the analyzed stages are marked by arrows. [file 12862_2020_1680_MOESM18_ESM.pdf]

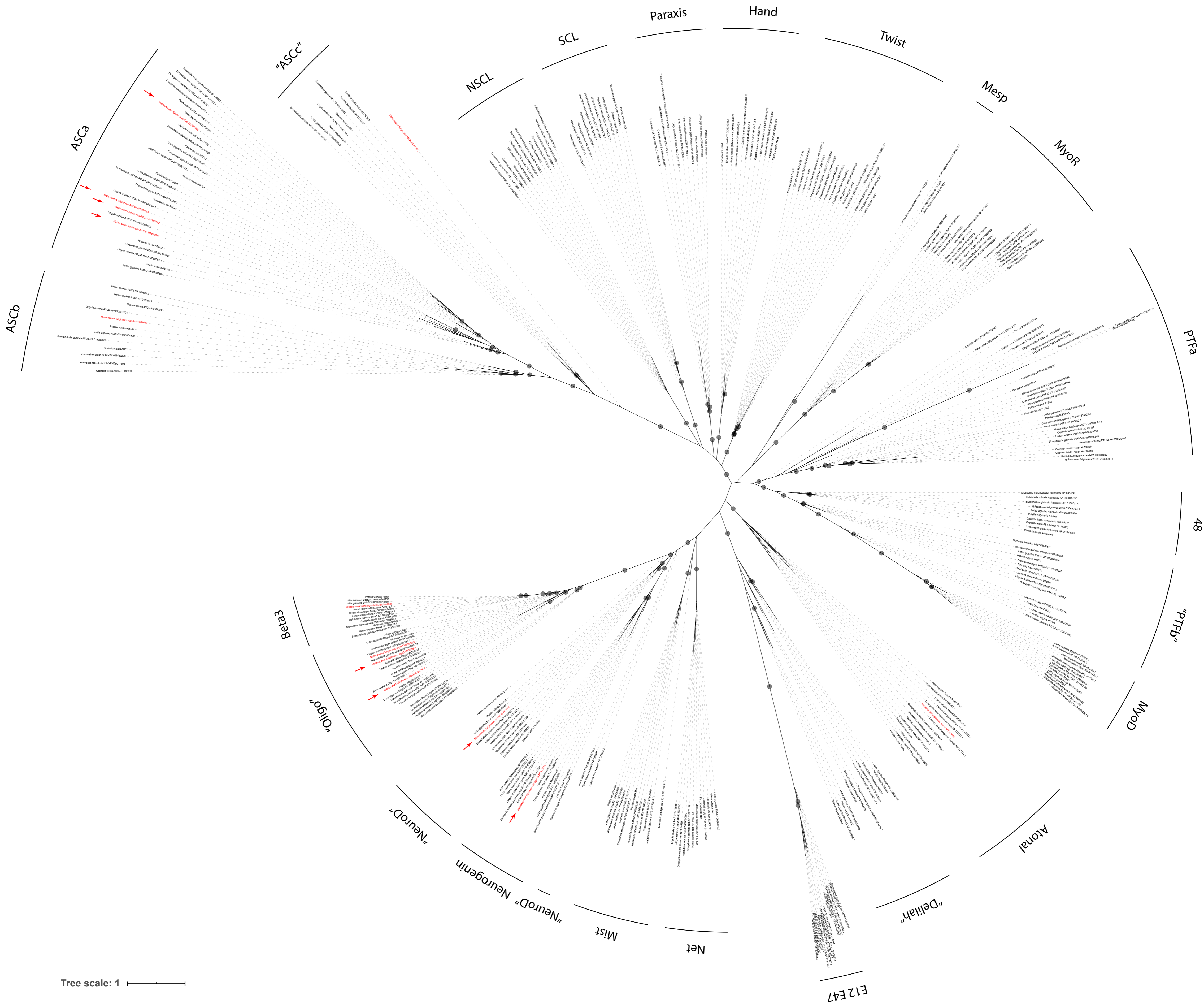

Supplement: Supplementary file 20 — Additional file 20 Evolution of bHLH group A genes. Adobe Acrobat file (.pdf). Unrooted maximum-likelihood tree (IQ-TREE, model LG + R6 chosen by Modelfinder). Branches with approximate Bayes test ≥0.98 are labelled. Sequences of M. fuliginosus are highlighted in red. Genes we found being expressed in the analyzed stages are marked by arrows. [file 12862_2020_1680_MOESM20_ESM.pdf]

SoxC

SoxE

SoxF

SoxD

SoxB

Tree scale: 1

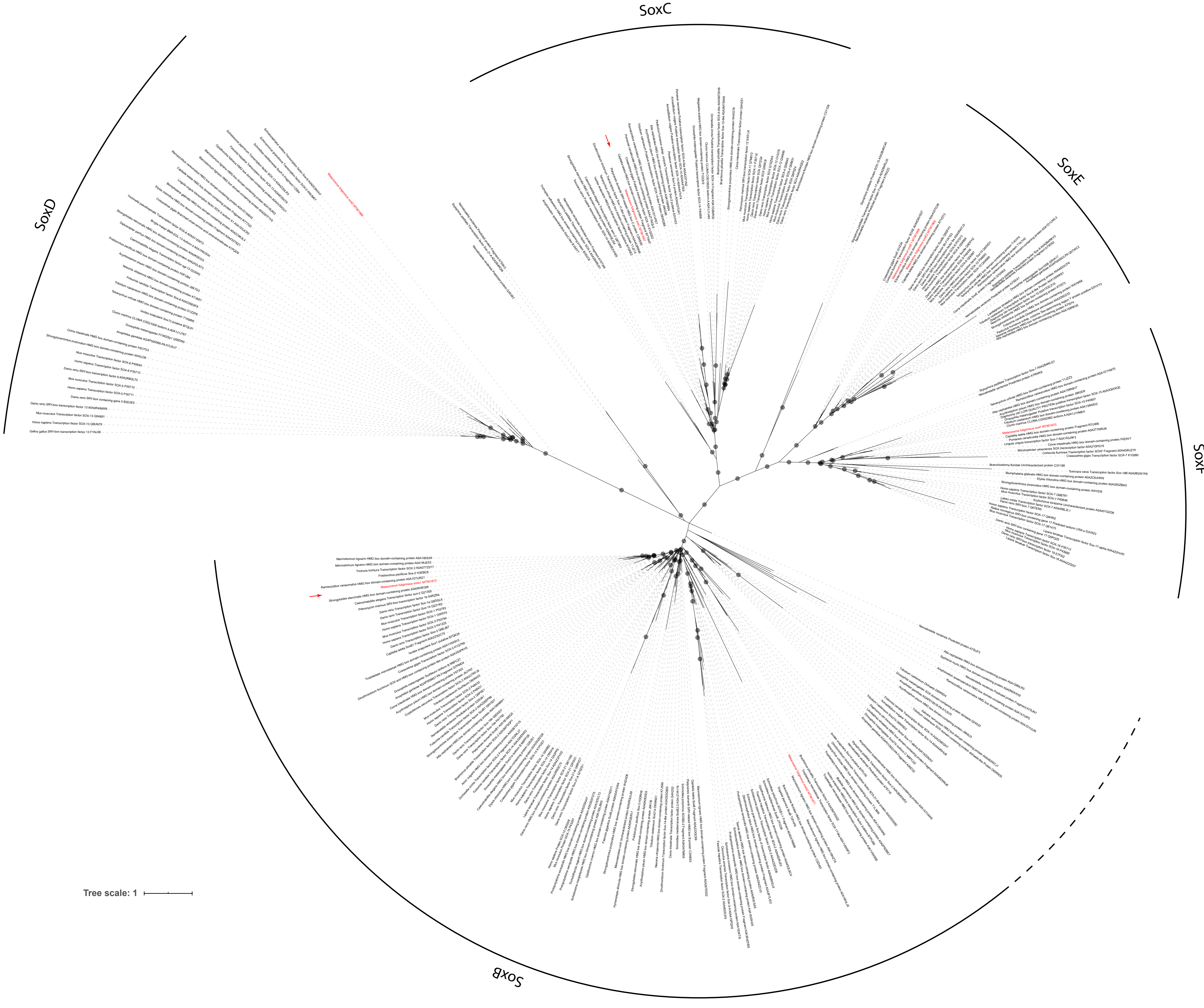

Supplement: Supplementary file 22 — Additional file 22 Evolution of Sox genes. Adobe Acrobat file (.pdf). Unrooted maximum-likelihood tree (IQ-TREE, model LG + R8 chosen by Modelfinder). Branches with approximate Bayes test ≥0.98 are labelled. Sequences of M. fuliginosus are highlighted in red. Genes we found being expressed in the analyzed stages are marked by arrows. [file 12862_2020_1680_MOESM22_ESM.pdf]

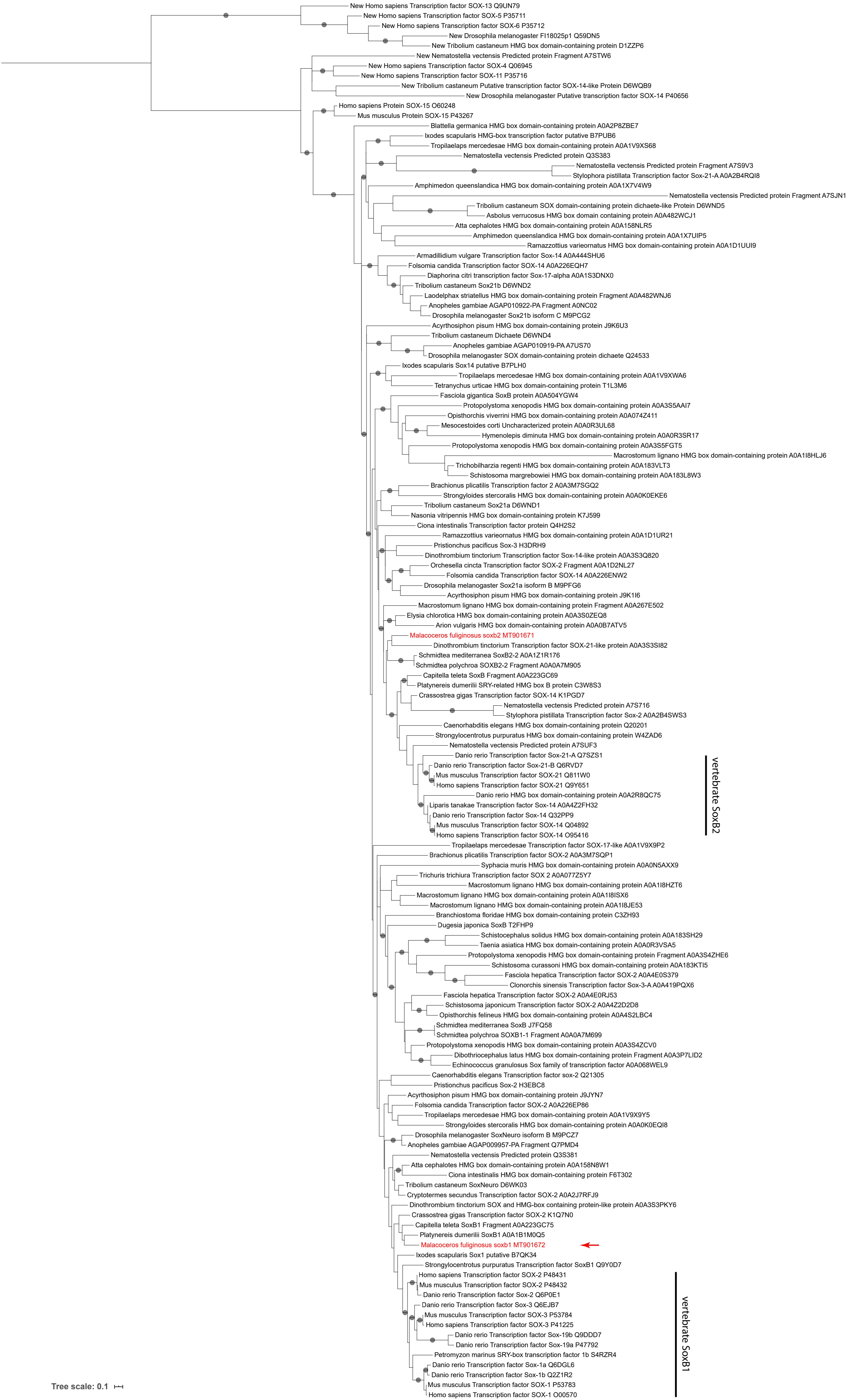

Tree scale: 0.1

SoxD

SoxC

SoxB2

SoxB1

vertebrate SoxB2

vertebrate SoxB1

Supplement: Supplementary file 23 — Additional file 23 Evolution of SoxB genes. Adobe Acrobat file (.pdf). The tree is based on a subset of the analysis shown in Fig. S6 with a SoxB-specific sequence and longer alignment. Maximum-likelihood tree (IQ-TREE, model JTT + R5 chosen by Modelfinder). Branches with approximate Bayes test ≥0.98 are labelled. Sequences of M. fuliginosus are highlighted in red. Genes we found being expressed in the analyzed stages are marked by arrows. [file 12862_2020_1680_MOESM23_ESM.pdf]

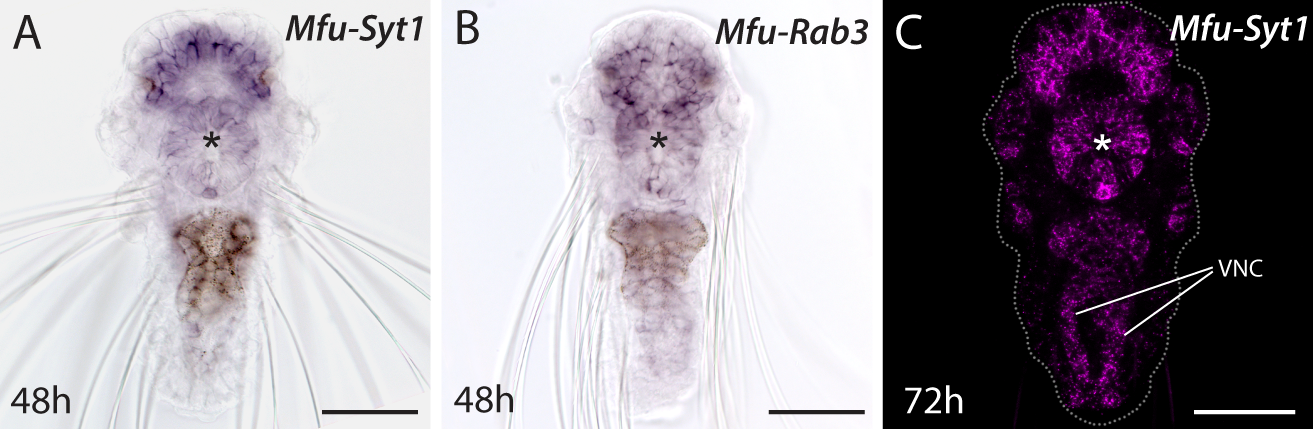

Supplement: Supplementary file 24 — Additional file 24 Expression of synaptic genes Mfu-Syt1 and Mfu-Rab3 in stages 48–72 hpf. Image file (*.tif). (A,B) WMISH of Mfu-Syt1 and Mfu-Rab3 at 48 hpf. (C) FISH of Mfu-Syt1 at 72 hpf showing the expression in the VNC. Scale bars: 50 μm. [file 12862_2020_1680_MOESM24_ESM.tif]

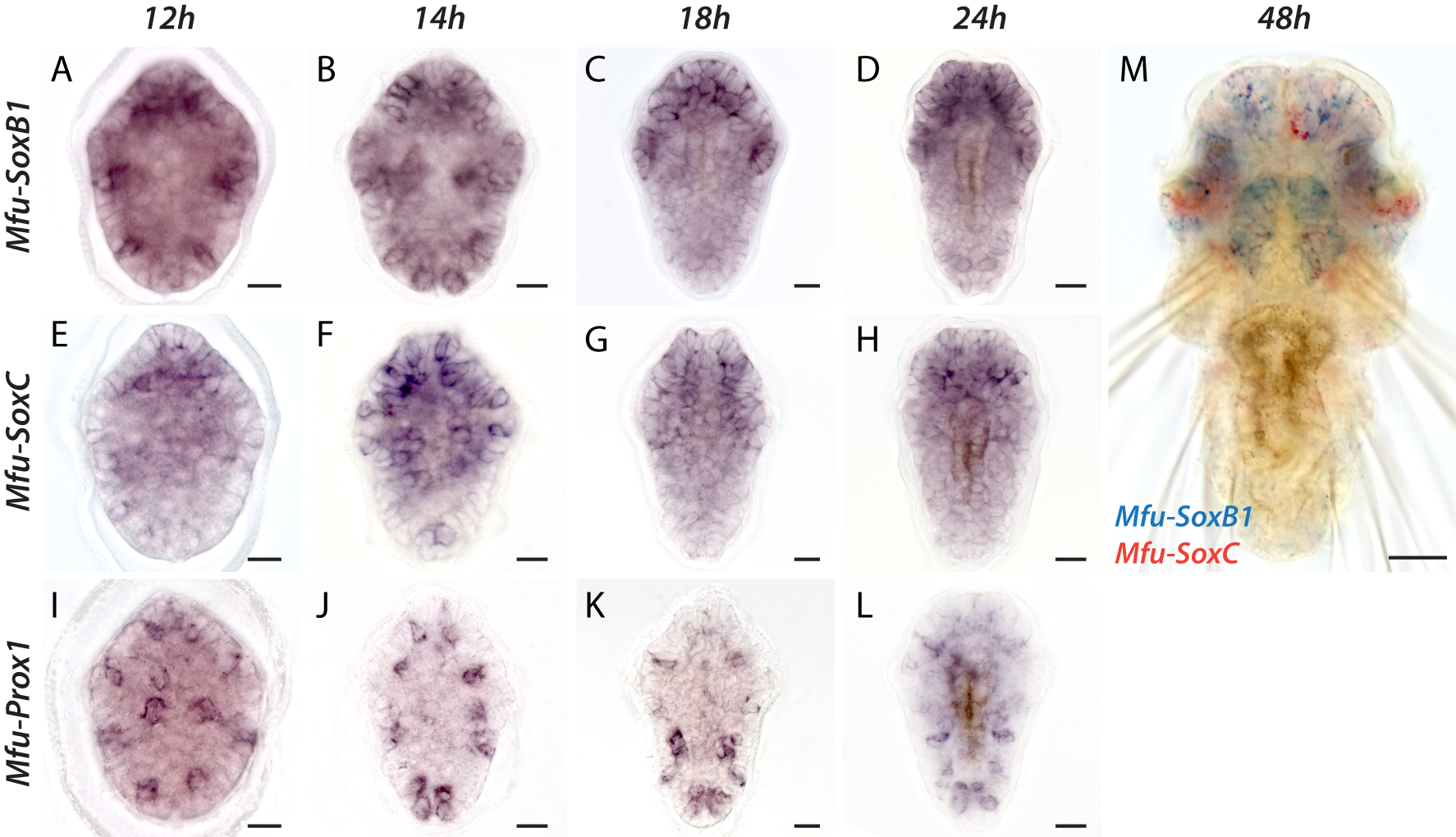

Supplement: Supplementary file 25 — Additional file 25 Expression of Mfu-SoxB1, Mfu-SoxC, and Mfu-Prox1 from 12 hpf onwards. Image file (*.tif). (A-L) WMISH in stages 12–24 hpf. (M) Double ISH of Mfu-SoxB1 and Mfu-SoxC at 48 hpf. Scale bars: 20 μm. [file 12862_2020_1680_MOESM25_ESM.tif]

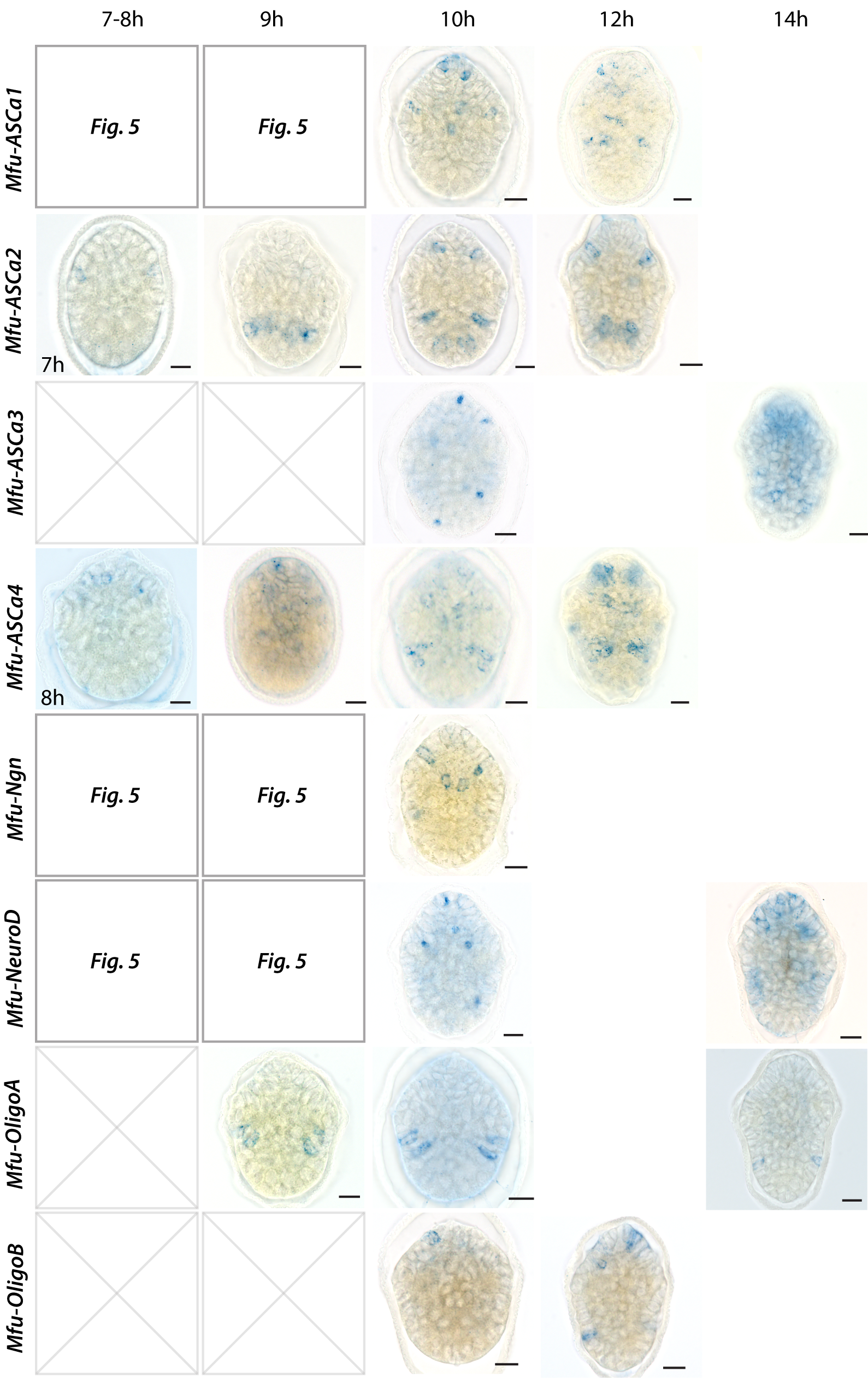

Supplement: Supplementary file 26 — Additional file 26. Expression of proneural bHLH genes in stages 7–14 hpf. Image file (*.tif). Scale bars: 20 μm. [file 12862_2020_1680_MOESM26_ESM.tif]
