# Supplementary material for: The development of early pioneer neurons in the annelid Malacoceros fuliginosus
Source: BMC Evol Biol. 2020 Sep 14;20:117. doi: 10.1186/s12862-020-01680-x (PMC7489019; doi:10.1186/s12862-020-01680-x)
Supplement: Supplementary file 19 — Additional file 19 Evolution of Prox1 genes. Adobe Acrobat file (.pdf). Unrooted maximum-likelihood tree (IQ-TREE, model LG + R4 chosen by Modelfinder). Branches with approximate Bayes test ≥0.98 are labelled. Sequences of M. fuliginosus are highlighted in red. Genes we found being expressed in the analyzed stages are marked by arrows. [file 12862_2020_1680_MOESM19_ESM.pdf]

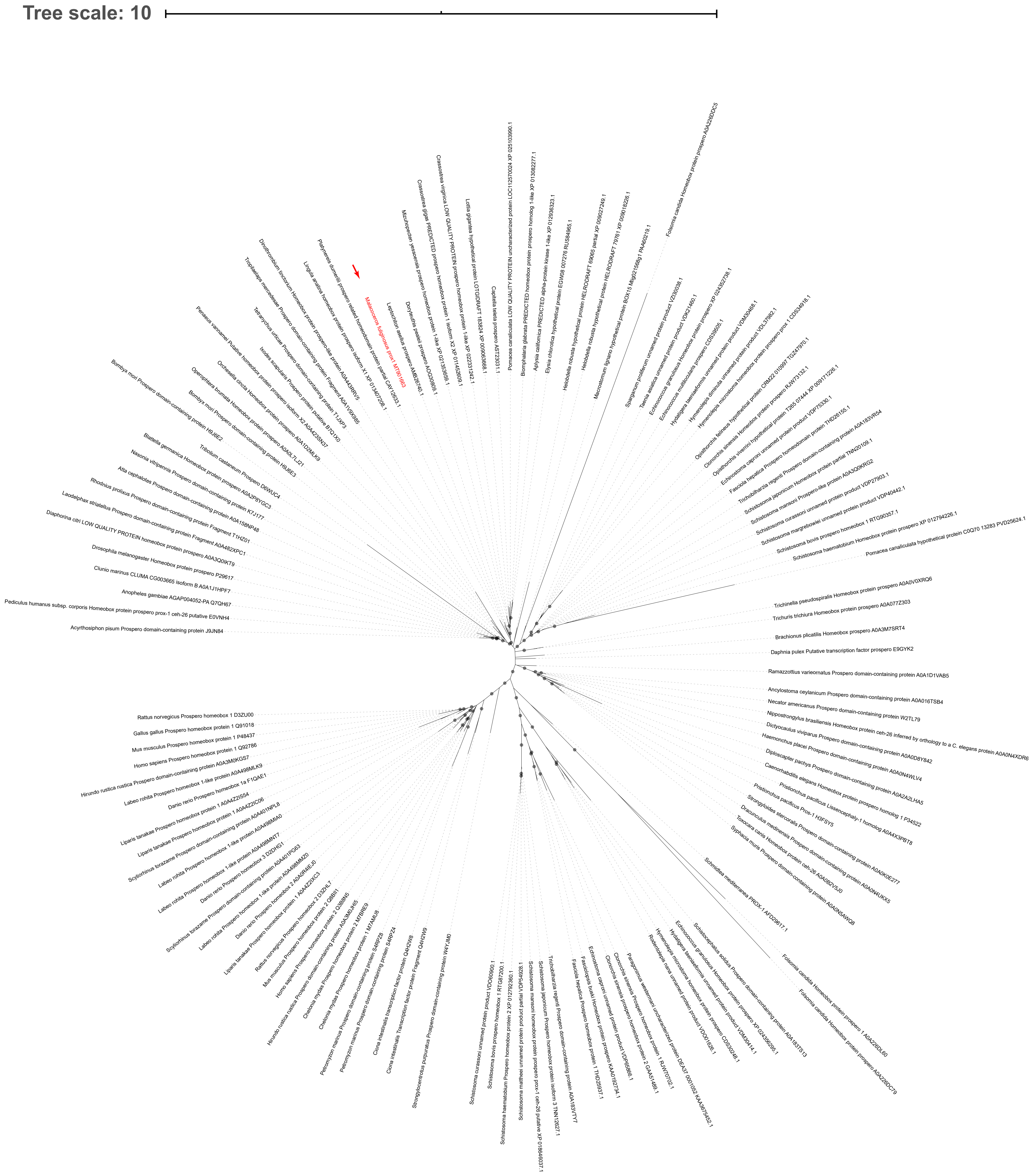
Tree scale: 10
Acyrthosiphon pisum Prospero domain-containing protein J9JN84
Pediculus humanus subsp. corporis Homeobox protein prospero prox-1 ceh-26 putative E0VNH4
Anopheles gambiae AGAP004052-PA Q7QH67
Clunio marinus CLUMA CG003665 isoform B A0A1J1HPF7
Drosophila melanogaster Homeobox protein prospero P29617
Diaphorina citri LOW QUALITY PROTEIN homeobox protein prospero A0A3Q0IKT9
Laodelphax striatellus Prospero domain-containing protein Fragment A0A482XPC1
Rhodnius prolixus Prospero domain-containing protein Fragment T1HZ01
Atta cephalotes Prospero domain-containing protein A0A158NP48
Nasonia vitripennis Prospero domain-containing protein K7J177
Blattella germanica Homeobox protein prospero A0A2P8YGC3
Tribolium castaneum Prospero D6WUC4
Bombyx mori Prospero domain-containing protein H9JIE2
Bombyx mori Prospero domain-containing protein H9JIE3
Operophtera brumata Homeobox protein prospero A0A0L7LJ21
Orchesella cincta Homeobox protein prospero A0A1D2MLK9
Penaeus vannamei Putative homeobox protein prospero isoform X2 A0A423SN37
Ixodes scapularis Prospero protein putative B7Q1X0
Tetranychus urticae Prospero domain-containing protein T1JXP3
Tropilaelaps mercedesae Prospero domain-containing protein Fragment A0A1V9XB85
Dinothrombium tinctorium Homeobox protein prospero-like protein A0A443RRV5
Lingula anatina homeobox protein prospero isoform X1 XP 013407208.1
Platynereis dumerilii prospero related homeodomain protein partial CAY12633.1
Malacoceros fuliginosus prox1 MT901663
Leptochiton asellus prospero AMB26740.1
Doryteuthis pealeii prospero AOG30809.1
Mizuhopecten yessoensis prospero homeobox protein 1-like XP 021353659.1
Crassostrea gigas PREDICTED prospero homeobox protein 1 isoform X2 XP 011452609.1
Crassostrea virginica LOW QUALITY PROTEIN prospero homeobox protein 1-like XP 022331242.1
Lottia gigantea hypothetical protein LOTGIDRAFT 183824 XP 009063668.1
Capitella teleta prospero ASTZ3031.1
Pomacea canaliculata LOW QUALITY PROTEIN uncharacterized protein LOC112570024 XP 025103990.1
Biomphalaria glabrata PREDICTED homeobox protein prospero homolog 1-like XP 013082277.1
Aplysia californica PREDICTED alpha-protein kinase 1-like XP 012936323.1
Elysia chlorotica hypothetical protein EGW08 007276 RUS84965.1
Helobdella robusta hypothetical protein HELRODRAFT 69065 partial XP 009027249.1
Helobdella robusta hypothetical protein HELRODRAFT 79761 XP 009018226.1
Macrostomum lignano hypothetical protein BOX15 Mlig021589g1 PAA69219.1
Folsomia candida Homeobox protein prospero A0A226DDC5
Spargianum proliferum unnamed protein product VZI30038.1
Taenia asiatica unnamed protein product VDK21460.1
Echinococcus granulosus Homeobox protein prospero XP 024352738.1
Echinococcus multilocularis prospero CDS35605.1
Hydatigera taeniaeformis unnamed protein product VDM30468.1
Hymenolepis diminuta unnamed protein product VDL37982.1
Hymenolepis microstoma homeobox protein prospero prox 1 CDS34918.1
Opisthorchis felineus hypothetical protein CRM22 010997 TGZ47970.1
Clonorchis sinensis Homeobox protein prospero RJW73132.1
Opisthorchis viverrini hypothetical protein T265 07444 XP 009171226.1
Echinostoma caproni unnamed protein product VDP75330.1
Fasciola hepatica Prospero homeodomain protein THD26155.1
Trichobilharzia regenti Prospero domain-containing protein A0A183VR64
Schistosoma japonicum Homeobox protein partial TNN20109.1
Schistosoma mansoni Prospero-like protein A0A3Q0KRG2
Schistosoma curassoni unnamed protein product VDP27903.1
Schistosoma margrebowiei unnamed protein product VDP40442.1
Schistosoma bovis prospero homeobox 1 RTG90357.1
Schistosoma haematobium Homeobox protein prospero XP 012794226.1
Pomacea canaliculata hypothetical protein C0Q70 13283 PVD25624.1
Trichinella pseudospiralis Homeobox protein prospero A0A0V0XRQ6
Trichuris trichiura Homeobox protein prospero A0A077Z303
Brachionus plicatilis Homeobox prospero A0A3M7SRT4
Daphnia pulex Putative transcription factor prospero E9GYK2
Ramazzottius varieornatus Prospero domain-containing protein A0A1D1VAB5
Ancylostoma ceylanicum Prospero domain-containing protein A0A016TSB4
Necator americanus Prospero domain-containing protein W2TL79
Nippostrongylus brasiliensis Homeobox protein ceh-26 inferred by orthology to a C. elegans protein A0A0N4XDR6
Dictyocaulus viviparus Prospero domain-containing protein A0A0D8Y842
Haemonchus placei Prospero domain-containing protein A0A0N4WLV4
Diploscapter pachys Prospero domain-containing protein A0A2A2LHA5
Caenorhabditis elegans Homeobox protein prospero homolog 1 P34522
Pristionchus pacificus Pros-1 H3FSY5
Pristionchus pacificus Lissencephaly-1 homolog A0A4X3PBT8
Strongyloides stercoralis Prospero domain-containing protein A0A0K0E277
Dracunculus medinensis Prospero domain-containing protein A0A0N4UKX5
Toxocara canis Homeobox protein ceh-26 A0A0B2VSJ0
Syphacia muris Prospero domain-containing protein A0A0N5AWQ8
Schmidtea mediterranea PROX-1 AFD29617.1
Folsomia candida Homeobox protein prospero 1 A0A226DL60
Folsomia candida Homeobox protein prospero A0A226DC79
Schistocephalus solidus Prospero domain-containing protein A0A183T513
Echinococcus granulosus Homeobox protein prospero XP 024356295.1
Hydatigera taeniaeformis unnamed protein product VDM30414.1
Hymenolepis microstoma homeobox protein prospero CDS30248.1
Rodentolepis nana unnamed protein product VDO01636.1
Paragonimus westermani uncharacterized protein DEA37 0001052 KAA3675452.1
Clonorchis sinensis Prospero homeobox protein 1 RJW70702.1
Clonorchis sinensis prospero homeobox protein 2 GAA51489.1
Echinostoma caproni unnamed protein product VDP65866.1
Fasciolopsis buski Homeobox protein prospero KAA0192734.1
Fasciola hepatica Prospero homeobox protein 1 THD25937.1
Trichobilharzia regenti Prospero domain-containing protein A0A183VT77
Schistosoma japonicum Prospero homeobox protein isoform 3 TNN12627.1
Schistosoma mansoni homeobox protein prospero prox-1 ceh-26 putative XP 018646037.1
Schistosoma mattheei unnamed protein product partial VDP54928.1
Schistosoma haematobium Prospero homeobox protein 2 XP 012792360.1
Schistosoma bovis prospero homeobox 1 RTG87200.1
Schistosoma curassoni unnamed protein product VDO69900.1
Strongylocentrotus purpuratus Prospero domain-containing protein W4YJM0
Ciona intestinalis Transcription factor protein Fragment Q4H2W9
Ciona intestinalis transcription factor protein Q4H2W8
Petromyzon marinus Prospero domain-containing protein S4RPZ4
Petromyzon marinus Prospero domain-containing protein S4RPZ8
Chelonia mydas Prospero homeobox protein 1 M7AMJ8
Chelonia mydas Prospero homeobox protein 2 M7BRE9
Hirundo rustica rustica Prospero domain-containing protein A0A3M0JHI5
Homo sapiens Prospero homeobox protein 2 Q3B8N5
Mus musculus Prospero homeobox protein 2 Q8BII1
Rattus norvegicus Prospero homeobox 2 D3ZHL7
Liparis tanakae Prospero homeobox protein 1 A0A4Z2IXC3
Danio rerio Prospero homeobox 2 A0A0R4IEJ0
Labeo rohita Prospero homeobox 1-like protein A0A498MMZ0
Scyliorhinus torazame Prospero domain-containing protein A0A401PG63
Danio rerio Prospero homeobox 3 D2DHG1
Labeo rohita Prospero homeobox 1-like protein A0A498MNT7
Labeo rohita Prospero homeobox 1-like protein A0A498MIA0
Scyliorhinus torazame Prospero domain-containing protein A0A401NPL8
Liparis tanakae Prospero homeobox protein 1 A0A4Z2IC06
Liparis tanakae Prospero homeobox protein 1 A0A4Z2IS54
Danio rerio Prospero homeobox 1a F1QAE1
Labeo rohita Prospero homeobox 1-like protein A0A498MLK9
Hirundo rustica rustica Prospero domain-containing protein A0A3M0KGS7
Homo sapiens Prospero homeobox protein 1 Q92786
Mus musculus Prospero homeobox protein 1 P48437
Gallus gallus Prospero homeobox protein 1 Q91018
Rattus norvegicus Prospero homeobox 1 D3ZU00
