# Supplementary material for: The development of early pioneer neurons in the annelid Malacoceros fuliginosus
Source: BMC Evol Biol. 2020 Sep 14;20:117. doi: 10.1186/s12862-020-01680-x (PMC7489019; doi:10.1186/s12862-020-01680-x)
Supplement: Supplementary file 21 — Additional file 21 Evolution of achaete scute (ASC) genes. Adobe Acrobat file (.pdf). The tree is based on a subset of the analysis shown in Fig. S4 with an ASC-specific and longer alignment. Maximum-likelihood tree (IQ-TREE, model JTTDCMut+R6 chosen by Modelfinder). Branches with approximate Bayes test ≥0.98 are labelled. Sequences of Malacoceros fuliginosus are highlighted in red, sequences of Capitella teleta in green, of Platynereis dumerilii in blue and of Nematostella vectensis in yellow. Genes we found being expressed in the analyzed stages are marked by red arrows. Genes of C. teleta, P. dumerilii, and N. vectensis, which are reported to be expressed in the context of nervous system development are also marked by arrows. Dashed arrows are used for sequences which are also broadly expressed in other tissues. For genes of P. dumerilii the expression level is indicated based on data in PdumBase. [file 12862_2020_1680_MOESM21_ESM.pdf]

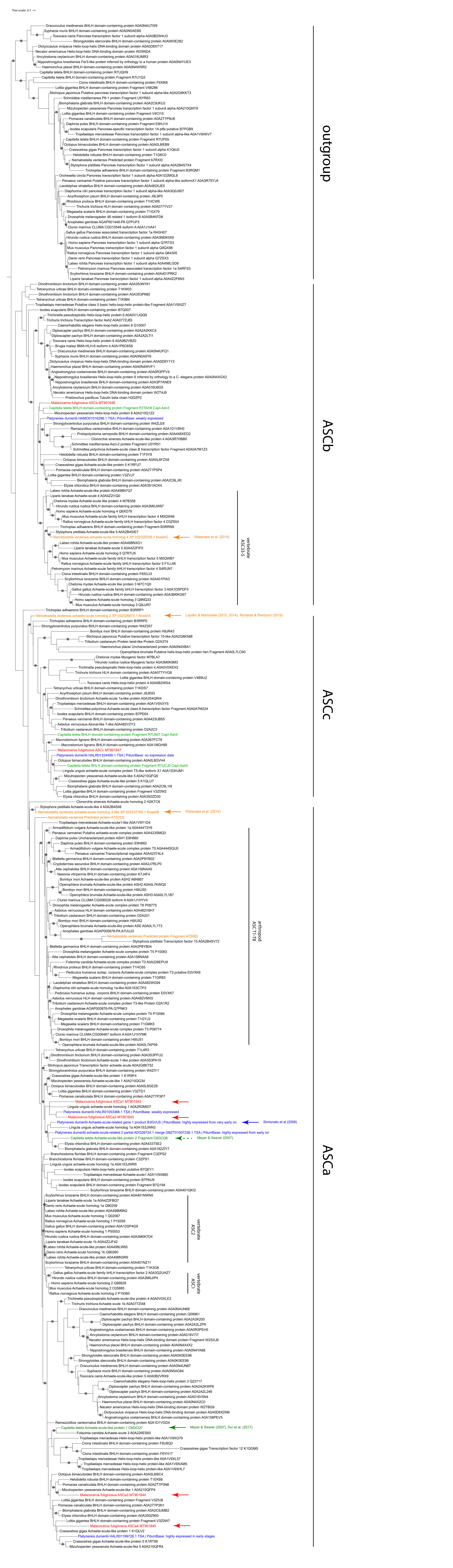
Tree scale: 0.1
outgroup
ASCb
vertebrate ASC3-5
Watanabe et al. (2014)
ASCc
Layden & Martindale (2012, 2014), Richards & Rentzsch (2015)
Watanabe et al. (2014)
arthropod ASC T1-T8
ASCa
Simionato et al (2008)
Meyer & Seaver (2007)
vertebrate ASC2
vertebrate ASC1
Meyer & Seaver (2007), Sur et al. (2017)
